# A Neuronal Activity-Dependent Dual Function Chromatin-Modifying Complex Regulates *Arc* Expression^1^,^2^,^3^

**DOI:** 10.1523/ENEURO.0020-14.2015

**Published:** 2015-03-14

**Authors:** Nicodemus E. Oey, How Wing Leung, Rajaram Ezhilarasan, Lei Zhou, Roger W. Beuerman, Hendrika M.A. VanDongen, Antonius M.J. VanDongen

**Affiliations:** 1 Program in Neuroscience and Behavioral Disorders, Duke-NUS Graduate Medical School, Singapore, 169857, Singapore; 2 Singapore Eye Research Institute, Singapore, 169856

**Keywords:** chromatin modification, epigenetics, histone acetylation, immediate early gene, neuronal activity, transcription

## Abstract

Chromatin modification is an important epigenetic mechanism underlying neuroplasticity. Histone methylation and acetylation have both been shown to modulate gene expression, but the machinery responsible for mediating these changes in neurons has remained elusive. Here we identify a chromatin-modifying complex containing the histone demethylase PHF8 and the acetyltransferase TIP60 as a key regulator of the activity-induced expression of Arc, an important mediator of synaptic plasticity. Clinically, mutations in PHF8 cause X-linked mental retardation while TIP60 has been implicated in the pathogenesis of Alzheimer’s disease. Within minutes of increased synaptic activity, this dual function complex is rapidly recruited to the Arc promoter, where it specifically counteracts the transcriptionally repressive histone mark H3K9me2 to facilitate the formation of the transcriptionally permissive H3K9acS10P, thereby favoring transcriptional activation. Consequently, gain-of-function of the PHF8−TIP60 complex in primary rat hippocampal neurons has a positive effect on early activity-induced *Arc* gene expression, whereas interfering with the function of this complex abrogates it. A global proteomics screen revealed that the majority of common interactors of PHF8 and TIP60 were involved in mRNA processing, including PSF, an important molecule involved in neuronal gene regulation. Finally, we proceeded to show, using super-resolution microscopy, that PHF8 and TIP60 interact at the single molecule level with PSF, thereby situating this chromatin modifying complex at the crossroads of transcriptional activation. These findings point toward a mechanism by which an epigenetic pathway can regulate neuronal activity-dependent gene transcription, which has implications in the development of novel therapeutics for disorders of learning and memory.

## Abstract (precis)

The regulation of neuronal gene expression requires dynamic changes in chromatin structure as evidenced by the fact that dysregulation of the enzymes responsible for chromatin modification often leads to intellectual disability. In this article, we characterize a chromatin-modifying complex containing the X-linked mental retardation-associated protein PHF8 and the Alzheimer’s disease-associated protein TIP60, which regulates the expression of an important neuronal activity-dependent gene, *Arc*.

## Significance Statement

The regulation of neuronal gene expression requires dynamic changes in chromatin structure as evidenced by the fact that dysregulation of the enzymes responsible for chromatin modification often leads to intellectual disability. In this article, we characterize a chromatin-modifying complex containing the X-linked mental retardation-associated protein PHF8 and the Alzheimer’s disease-associated protein TIP60, which regulates the expression of an important neuronal activity-dependent gene, *Arc*. By interfering with the enzymatic function of this complex, we show that it is possible to alter the ability of neurons to induce transcription in response to synaptic activity. This work supports an enzymatic mechanism for the epigenetic control of neuronal transcriptional programs with implications in the possible development of novel therapeutics for disorders of learning and memory.

## Introduction

Activity-dependent transcription of effector genes, a prerequisite for memory formation (Tzingounis and Nicoll, 2006), is a highly complex process (Inoue et al., 2010; West and Greenberg, 2011). Several epigenetic mechanisms have been put forth to explain the remarkable ability of neurons to dynamically regulate gene expression, including chromatin modification, which is capable of altering gene expression programs and even induce alternative splicing (Day and Sweatt, 2011). The importance of chromatin modification in learning and memory is demonstrated by the fact that dysfunction of chromatin-modifying enzymes causes severe memory impairment, which ranges from Alzheimer’s disease to intellectual disability (Lewis et al., 1981; Lubin et al., 2011; Ronan et al., 2013). Recent evidence has shown that the rapid induction of immediate-early genes, such as *Arc* (activity-regulated cytoskeletal-associated protein), in response to neuronal activity is mediated by a mechanism involving the escape of promoter-proximal RNA polymerase II into transcriptional elongation (Kim et al., 2010; Saha et al., 2011). The idea that stimulus-dependent rapid gene induction is controlled at the level of transcriptional elongation and mRNA processing is conserved across many cell types and is likely to be mediated by modification to chromatin structure (Hargreaves et al., 2009). Both the acetylation and methylation of histones have been purported to be important in activity-dependent gene transcription (Gupta-Agarwal et al., 2014; Lopez-Atalaya and Barco, 2014; Sen, 2014). Nevertheless, although it is known that enzymes are likely responsible for the chromatin modifications that contribute to neuronal gene activation, the nature of these epigenetic regulators is still obscure.

Here we report that the histone demethylase PHF8 cooperates with the acetyltransferase TIP60 in an activity-dependent manner to enable the rapid induction of the immediate-early gene *Arc* by specifically regulating H3K9acS10P, a dual-chromatin mark that is required for transcriptional activation. As no direct interaction between a demethylase and an acetyltransferase has yet been reported, we focused on precisely characterizing the localization of PHF8 and TIP60 using multi-color super-resolution microscopy and investigated their physical interaction using coimmunoprecipitation and proximity *in situ* ligation. Within minutes of neural network activation, we found that the complex containing PHF8 and TIP60 specifically upregulated the transcriptional-elongation associated mark H3K9acS10P, which is required for rapid gene induction through a mechanism that likely involves transcriptional elongation. Upon verifying that this complex is able to regulate the methylation and acetylation of transcriptionally active H3K4me3-positive histones, we examined the PHF8−TIP60 interactome through immunoprecipitation followed by mass spectrometry, which revealed that the majority of PHF8 and TIP60 interacting partners are indeed involved in transcription and RNA processing. Overexpression of PHF8, but not the inactive mutant PHF8−F279S (Koivisto et al., 2007), increased neuronal H3K9acS10P and *Arc* expression, whereas RNAi-mediated knockdown of PHF8 inhibited both activity-induced H3K9acS10P and *Arc*. Furthermore, both PHF8 and TIP60 were found to be recruited to the *Arc* promoter within minutes of neuronal activation. Finally, using single-molecule imaging techniques, we demonstrate that the two chromatin-modifying enzymes have a well-defined three-dimensional spatial relationship with each other, with each molecule occupying long-stranded structures that are closely associated with their common binding partner, polypyrimidine tract-binding protein (PTB) associated splicing factor (PSF), at the nucleosomal scale. The direct interaction between the chromatin modifier PHF8 with PSF, a long-term memory-associated splicing factor (Antunes-Martins et al., 2007; Kim et al., 2011) lends further evidence to the role of chromatin modification in transcriptional activation and cotranscriptional splicing in neuronal activity-dependent gene regulation.

## Materials and Methods

### Constructs and cloning

Full-length PHF8 was cloned through reverse transcriptase reaction of human brain cDNA (Marathon), and confirmed via Sanger sequencing against a construct of FLAG−PHF8, which was a generous gift from Petra de Graaf (Fortschegger et al., 2010). Fusion fluorescent constructs PHF8−mTurquoise2, PHF8−YFP, and PHF8−tdTomato were cloned by inserting the full-length PHF8 PCR product flanked by SalI and AgeI sites in-frame into the multiple cloning sites of the respective vectors. To generate PHF8−FLAG and TIP60−FLAG, the YFP sequence was excised with NotI and an oligonucleotide encoding the FLAG peptide sequence was annealed and ligated to the C-terminus of PHF8 and TIP60. Mutagenesis of PHF8 into PHF8−F279S was performed using the megaprimer method (Bloomer et al., 2007) by first performing a PCR with a reverse primer containing the single nucleotide mutation (c.836C>T) to generate a forward primer for a second PCR reaction amplifying the full-length gene.

### Neuronal cell culture

Hippocampi and cortices from E18 Sprague-Dawley rats of either sex were dissected aseptically, digested using a papain dissociation system (Worthington Biochemical), and cultured in media supplemented with B27 (Brewer and Price, 1996). The appropriate density of neurons were plated on poly-D-lysine-coated glass-bottom culture dishes (MatTek), eight-well Lab-Tek II chambered cover-glass (Nunc), or 96-well glass-bottom plates (Nunc) that had been double-coated with poly-D-lysine overnight. Neurons were fed weekly by replacing half of the medium. HEK293, HeLa, and U2OS cells were obtained from the author’s University Cell Culture Facility, and were cultured in high-glucose DMEM (Gibco) with 10% fetal bovine serum (Invitrogen) and 1% penicillin−streptomycin. Cells were plated on the poly-D-lysine coated glass-bottom dishes for imaging or the Lab-Tek II chambered cover-glasses for super-resolution imaging.

### Transfections and neuronal stimulations

Primary neurons were transfected between DIV 12 and 21 as previously described, with a few modifications (Van de Ven et al., 2005). Briefly, Lipofectamine:DNA complexes were formed in a suitable amount of Neuronal Transfection Media (BrainBits) for 15 min at room temperature (RT). Neuronal growth medium was aspirated and the complexes were added to the neurons for 15 min, after which the neuronal medium was restored. HEK293, U2OS, and HeLa cells were transfected similarly, except that DMEM with high-glucose media was used and the Lipofectamine 2000/DNA mixture was added directly to existing media. Each well in a six-well plate was transfected with a 2:1 ratio of transfection reagent to plasmid DNA. To stimulate synaptic NMDA receptors and network activity (Hardingham et al., 2001), a combination of 4-aminopyridine (4AP), bicuculline (Bic), and forskolin at final concentrations of 100 μM, 50 μM, and 50 μM, respectively, were added to the medium for the appropriate amount of time. In control wells, the same volume of vehicle (DMSO) was added to neurons before lysates were collected.

### Immunofluorescence

For construct coexpression experiments, transfected neuron/HEK293 cells were fixed with a solution containing 4% paraformaldehyde (PFA), 4% sucrose, and 1× PBS for 15 min at 4°C. The cells were subsequently incubated with 1 μM DAPI for 10 min and preserved in 97% Thiodiethanol (TDE; Sigma). For immunostaining, cells were fixed with 100% MeOH at −20°C for 10 min. Neurons/HEK293 cells were blocked with a solution containing 10% goat or donkey serum, 2% bovine serum albumin, and 1× PBS for 1 h at RT, except when the goat-anti-TIP60 (K-17; Santa Cruz Biotechnology) antibody was used, in which case blocking was done with 10% horse serum in PBS-0.1% Triton X. The primary antibodies were incubated for 1 h at RT in a dilution buffer containing 1:1 block solution and PBS-Triton X solution at the following dilutions: mouse-anti-Arc (C7), 1:300 (Santa Cruz Biotechnology); goat-anti-TIP60 (K17), 1:300; rabbit-anti-TIP60, 1:300 (Novus Biologicals); rabbit-anti-H3K9acS10P, 1:300 (Abcam). Dishes were washed 5 × 10 min with PBS-Triton X and incubated with Alexa-Fluor 488, Alexa-Fluor 568, or Alexa-Fluor 647 conjugated secondary antibodies (Invitrogen), 1:1000 in dilution buffer for 1 h at RT. Washing was repeated as per the above for 5 × 10 min and dishes were then stained with 1 μM DAPI for 10 min to label DNA, followed by mounting in 10%, 25%, 50%, and finally 97% TDE.

### Proximity ligation in situ assay

Stably transfected HEK293 cells expressing PHF8−YFP were fixed with 4% PFA for 15 min at 4°C, and blocked and permeabilized using a solution containing 10% donkey serum and 0.5% Tween-20 at 37°C. Cells were incubated overnight with primary antibodies: mouse-anti-GFP (1:1000; Roche) and goat-anti-TIP60 (1:300; Santa Cruz Biotechnology) were used to detect PHF8 and TIP60, respectively. Cells were washed five times with buffer A (10 mM Tris, 150 mM NaCl, and 0.05% Tween-20) and then incubated for 2 h with secondary antibodies conjugated to PLA probes: Duolink II anti-mouse plus and Duolink II anti-goat minus were diluted in antibody diluent to a concentration of 1:5 (OLink Bioscience) at 37°C. After five more washes with buffer A at room temperature, hybridization was performed by incubating at 37°C with the ligation solution (Duolink II Ligase, 1:40) for exactly 30 min. Ligation was stopped by a wash step and detection of the amplified probe was done with the Duolink II Detection Reagents Kit (Red). After a final wash step of 15 min × 5 in buffer B (200 mM Tris and 100 mM NaCl), cells were mounted and imaged. Negative controls were obtained by transfecting the mutant PHF8 (F279S) and by repeating the procedure with no primary antibodies.

### Widefield imaging, calcium imaging, and data analysis

Fluorescence images were obtained using a motorized inverted wide-field epifluorescence microscope (Nikon Eclipse Ti-E), using 40× and 60× Plan-Apo oil objectives, with numerical apertures of 1.35 and 1.49, respectively. Motorized excitation and emission filter wheels (Ludl Electronics) fitted with a DAPI/CFP/YFP/DsRed quad filter set (#86010, Chroma) were used together with filter cubes for DAPI, CFP, YFP, and TxRed (Chroma) to select specific fluorescence signals. Z-stacks were obtained spanning the entire nucleus and out-of-focus fluorescence was removed using the AutoQuant deconvolution algorithm (Media Cybernetics). Calcium imaging was done either through a cell-permeable Ca^2+^-sensitive dye (Fluo-4 AM, Invitrogen) or the transfection of a genetically encoded Ca^2+^ sensor (gCamp6, medium isoform). Images were obtained in time series of 100 ms/frame, and quantification was performed through the Time Measurement feature of NIS Elements. For all purposes, Images were digitized using a cooled EM-CCD camera (iXon EM+ 885, Andor). Image acquisition was performed using NIS Elements AR 4.2 software (Nikon). NIS Elements Binary and ROI Analysis tools were used to segment nuclei based on DAPI signal intensity.

### Coimmunoprecipitation and Western blotting

Transfected HEK293 cells growing in six-well plates were allowed to express overnight at 37°C to yield >90% transfection efficiency. Throughout the entire procedure, cultures and subsequent lysates were kept on ice or at 4°C. For coimmunoprecipitation, the cultures were washed once with 1 ml of PBS, and lysed in 500 μl of lysis buffer for 30 min, then scraped into 1.5 ml tubes. Lysis buffer consisted of 5 mM HEPES pH 7.2, 0.5% NP40, 250 mM NaCl, 2 mM EDTA, 10% glycerol, 1:100 dilution of protease inhibitor cocktail (Sigma-Aldrich). The lysates were spun down for 20 min at 16,000 ×g to pellet cell debris. Then 500 μl of the supernatant was incubated on a rotator with 5 μl mouse-anti GFP (Ruiz et al., 2012) for 90 min, followed by 100 μl of Protein-A/G Plus-Agarose (Santa Cruz Biotechnology) for another 60 min on a rotator. The beads were spun down at 1000 ×g for 5 min and the supernatant was removed. Immunoprecipitation (IP) fractions were then washed and resuspended in 1 ml lysis buffer a total of three times. The beads and input lysates were resuspended and boiled at 95°C for 5 min in sample buffer, resolved by SDS-PAGE with Tris-glycine gels (Bio-Rad), transferred to 0.2 μm PVDF membranes (Invitrogen), and imunoblotted. The primary antibodies used were anti-GFP (mouse-monoclonal; Roche) and anti-FLAG (mouse-monoclonal; Sigma).

### Immunoprecipitation and mass spectrometry

Immunoprecipitation was performed as above, with a pre-clearing step using 5 μg of purified rabbit IgG/IP. Following the final wash step, beads were resuspended and heated to 65°C for 10 min and run on 4-20% gradient Tris-glycine gels (Bio-Rad) for 3 h at constant 80 V. Gels were stained for 1 h with the GelCode Blue Stain (Ma et al., 2004), and washed extensively with ddH2O overnight. Using a sterile scalpel, bands that were represented in the PHF8/TIP60 IP fraction but not in the control YFP IP fraction were excised and kept in clean 1.5 ml tubes then spun down at 1000 ×g for 5 min. Following reduction and alkylation, interacting proteins were trypsinized overnight at 37°C. Peptides were dried and resuspended in mass-spectrometry-compatible buffer, and analyzed with one-dimensional nanoLC-MS/MS (Dionex UltiMate 3000 nanoLC system coupled with AB Sciex TripleTOF 5600 system) for protein identification. The IPI human protein database (version 3.77) was searched using ProteinPilot (version 4.5, AB Sciex) and the identified hits were analyzed using DAVID (http://david.abcc.ncifcrf.gov) for gene ontology annotation.

### Chromatin-immunoprecipitation and Triton X-acetic acid-urea histone electrophoresis

Neurons were treated with chemLTP as above; HEK293 cells growing in six-well plates were transfected with PHF8, TIP60, or both, and allowed to express overnight at 37°C to yield >90% transfection efficiency. DNA-protein crosslinking was done *in situ* by adding 37% formaldehyde to growth medium to a final concentration of 1%, for 8 min at RT, after which the unreactive formaldehyde was quenched with 1.25 M glycine, and then washed 3× with PBS and collected. One million cells were lysed in 100 μl of chromatin immunoprecipitation (ChIP) lysis buffer (1%SDS with a 1:100 dilution of protease inhibitor cocktail; Sigma-Aldrich). Next, 10 μl of unsheared input chromatin were collected, and the remaining lysates were spun down for 10 min at 10,000 ×g, and then sonicated for eight cycles using the Bioruptor 2000 (Diagenode) at the high setting. Then, 10 μl of unsheared and sheared chromatin were loaded on a 1% agarose gel to visualize shearing efficiency. The sheared chromatin was then diluted with ChIP dilution buffer (0.01%SDS, 1.1% Triton X, 1.2 mM EDTA, 20 mM Tris-HCl pH 8.0, 150 mM NaCl), and DNA was quantified using a NanoDrop. Fifty micrograms of chromatin was incubated with 3 μl of the primary antibody rabbit-antiH3K4me3 (ActiveMotif) for 3 h, followed by the addition of 50 μl of Protein-A/G Plus-Agarose beads (Santa Cruz Biotechnology), which was incubated for 60 min on a rotator. The beads were spun down at 1000 ×g for 5 min and the unbound fraction removed. IP fractions were then washed and resuspended in three consecutive washes using the low-salt ChIP wash buffer, high-salt ChIP wash buffer, and LiCl ChIP wash buffer (Millipore), followed by two final washes in TE buffer. Immunoprecipitated chromatin was eluted, cross-links were reversed, and equal amounts of protein were loaded onto SDS-PAGE gels or Triton X-acetic acid-urea (TAU) gels, which were made fresh on the day of the experiment following published protocols (Shechter et al., 2007). Alternatively for ChIP-RT-PCR analyses, immunoprecipitated chromatin was eluted, reverse cross-linked, and treated with RNAse A for 1 h at 37°C and proteinase K for 8 h at 62°C. DNA was purified using a spin column (Qiagen), and eluted into 30 μl volumes, out of which 2 μl was used in qRT-PCR using primers against the transcriptional start site (TSS) of the rat *Arc* (NCBI Gene ID: 54323), *BDNF* (NCBI Gene ID: 24225), *Synaptophysin* (NCBI Gene ID: 24804), and *Fos* (NCBI Gene ID: 314322) genes. Control genes included *Rpl19* (NCBI Gene ID: 81767), *Txnip* (NCBI Gene ID: 117514), *Homer1A* (NBCI Gene ID: 29546), *JunB* (NCBI Gene ID: 24517), and *Gapdh* (NCBI Gene ID: 24383).

### 3D structured illumination microscopy (3D-SIM)

Neurons were treated and then fixed at the appropriate timepoints. Autofluorescence was reduced with sodium borohydride (0.1% NaBH_4_) for 5 min. Immunostaining was performed as described above, with a final wash step following secondary antibody incubation to transition the neurons into a TDE mounting medium. Serial dilutions of 10%, 25%, 50%, and finally 97% TDE, with a final refractive index of 1.512, which directly matches that of glass, was used to mount the samples. A Zeiss Elyra PS-1 super-resolution system equipped with 405, 488, 561, and 642 nm lasers (50 mW, 200 mW, 200 mW, and 150 mW, respectively) for excitation was used to acquire 3D-SIM images. A Zeiss 63X Plan-Apochromat (NA = 1.4) oil-immersion objective lens was used with a cooled EM-CCD camera (iXon EM+ 885; Andor) camera to capture a 1 megapixel field of view. Fifteen images per section per channel were acquired (made up of 3 rotations and 5 phase movements of the diffraction grating) at a z-spacing of 0.125 µm. Structured illumination reconstruction and alignment was completed using the Zen software (Zeiss).

### 3D stochastic optical reconstruction microscopy (3D-STORM)

Neurons cultured on LabTek II chambered cover-glass (thickness #1.5) were stimulated appropriately and fixed with 3% paraformaldehyde for 8 min, and autofluorescence was quenched with 0.1% NaBH_4_ for 5 min. After a blocking step, immunostaining was performed as described above, with antibody concentrations titrated to the appropriate dilution. After secondary antibody incubation, antibody−antigen complexes were post-fixed with 3% paraformaldehyde, 0.1% glutaraldehyde for 5 min, and then washed a total of 10 times in PBS. STORM imaging buffer containing cysteamine (Sigma) titrated with HCl to pH 7.4 and a cocktail of glucose oxidase and catalase solution was added to aliquots of 10% glucose in PBS. 3D-STORM imaging was done on a Nikon Ti microscope equipped with a 100× Apo-TIRF oil-immersion objective and an Andor iXon3 EM-CCD camera with the gain set at 200 mHz. A Coherent 488 nm, Sapphire 561 nm laser line operating at 150 mW (Coherent) and a 647 nm line with 180 mW output (Obis) were used to push the Atto-488, Alexa-568, and Alexa-647 fluorophores to the triplet state, in the presence of cysteamine HCl, glucose oxidase, and catalase, in a closed environmental chamber to prevent entry of molecular oxygen. Time lapses were acquired at 50 fps, for a total of at least 15,000 periods. Molecule list thresholding and 3D rendering were performed on the NIS Elements software with the STORM module installed (Nikon).

## Results

### The histone demethylase PHF8 colocalizes with the histone acetyltransferase TIP60 in the interchromatin space

**Figure 1:**
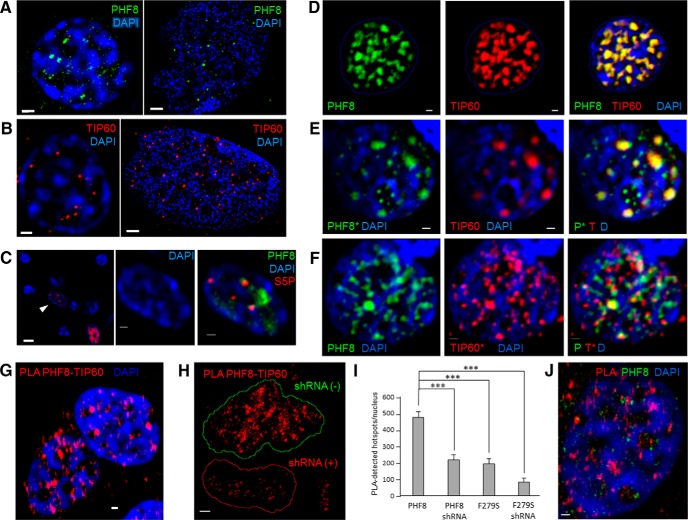
PHF8 and TIP60 colocalize and recruit each other in neuronal interchromatin space. ***A***, Endogenous PHF8 immunostained with anti-PHF8 antibody (ab36068; Abcam) forms hundreds of discrete puncta that specifically localize to the interchromatin space in hippocampal neurons (representative z-slice of a hippocampal neuronal nucleus; left, widefield; right, SIM). Scale bar, 1 μm. ***B***, Endogenous TIP60 forms puncta of roughly the same caliber as those of PHF8 above (left, hippocampal nucleus in widefield; right, SIM), which also localize to the interchromatin space. Scale bar, 1 μm. ***C***, A representative field of hippocampal neurons stained with an antibody against the phosphorylated CTD of RNA polymerase II (YSPTSPS phospho S5, abbreviated to S5P), showing that S5P, a marker of the transcription initiation complex, localizes to the same nuclear compartment as PHF8 in the nucleus. Scale bar, 1 μm. ***D,*** A hippocampal neuronal nucleus outlined in blue, showing the localization of spectrally distinct PHF8−tdTomato and TIP60−CFP pseudo-colored in green and red, respectively, which overlapped completely in the nuclear interchromatin space (merge channel, yellow pixels indicate colocalization). DAPI was used to stain the DNA (blue). Scale bar, 0.2 μm. ***E***, When TIP60 is overexpressed by itself in hippocampal neurons (middle, red), endogenous PHF8 (left, green) is seen to be recruited to the TIP60 puncta in hippocampal neurons (right, merge). DAPI was used to stain the DNA (blue). Scale bar, 0.2 μm. Asterisk (*) indicates endogenous protein staining. ***F***, When PHF8 is overexpressed by itself (left, green) in hippocampal neurons, endogenous TIP60 (middle) is seen to be recruited to the PHF8 puncta (right, merge). DAPI was used to stain the DNA (blue). Scale bar, 0.2 μm. Asterisk (*) indicates endogenous protein staining. ***G.*** Endogenous TIP60 is located within 30 nm of PHF8 as shown by P-LISA, showing distinct areas where PHF8 interacts with endogenous TIP60 (red spots) on the border with DAPI-dense regions (blue). Scale bar, 0.5 μm. ***H***, Two Hek293 nuclei are shown, one is positive for PHF8 shRNA (outlined in red) while the other is not (outlined in green). Positive PHF8−TIP60 interaction hotspots were stained as red punctae. Scale bar, 0.5 μm. ***I***, Quantification of the number of hotspots found in cells transfected with PHF8, PHF8 shRNA, the mutant PHF8 F279S, or, in F279S-transfected cells expressing PHF8, shRNA were quantified using Blobfinder, and the means and standard errors are displayed in a bar grapht (triple asterisks indicating statistical significance using the unpaired *t* test; *p* ≤ 0.0001). ***J***, Double immunofluorescence confirming the existence of PHF8 in the identified PLA hotspots where PHF8 and TIP60 interact. Scale bar, 0.5 μm.

In order to gain insight into their function, we first wanted to observe where the putative transcriptional coactivators reside in the neuronal nucleus. Endogenous and overexpressed PHF8 and TIP60 fused to spectrally non-overlapping fluorescent proteins eCFP, eYFP, mCherry, and tdTomato were imaged in primary hippocampal and cortical neurons. In all cases, PHF8 and TIP60 both localized to the nucleus, as expected (Fig. 1*A−F*). Importantly, however, both PHF8 and TIP60 formed hundreds of tiny punctate structures of similar calibre that localized to specific regions in the nucleus devoid of DAPI staining, indicative of areas known as the interchromatin space, where many nuclear processes are thought to occur (Politz et al., 1999; Tycon et al., 2014). Data from widefield microscopy is supported by the higher-resolution structured illumination microscopy, which shows that PHF8 and TIP60 localize to the interchromatin space, which are proposed locations of transcription factories (Eskiw et al., 2008). Indeed, immunostaining of the Ser5-phosphorylated C-terminal domain of RNA polymerase II (RNA Pol II) show that this interface is rich with transcriptional initiation-specific structures in primary neuronal nuclei (Fig. 1*C*). When expressed together in the same nucleus, we found that PHF8 tightly associated with TIP60 (Fig. 1*D*). This observation prompted us to ask whether the overexpression of PHF8 protein may cause recruitment of TIP60 to the PHF8 puncta, and vice versa. To this end, we transfected neurons either PHF8 or TIP60 singly and immunostained for endogenous TIP60 or PHF8, respectively, and found that exogenous TIP60 puncta were rich in endogenous PHF8 (Fig. 1*E*). Conversely, PHF8 puncta recruited endogenous TIP60 in hippocampal neurons (Fig. 1*F*). This bidirectional recruitment suggests that PHF8 and TIP60 physically interact.

In order to investigate this protein−protein interaction in greater detail, we used P-LISA (proximity ligation *in situ* assay), a technique that detects interactions between molecules that are obligatorily less than 30 nm from each other (Fredriksson et al., 2002). Using an antibody against TIP60 and an antibody against the GFP-epitope of PHF8−YFP, we performed P-LISA on HEK293 cells stably transfected with PHF8−YFP to detect specific PHF8−TIP60 interactions, which formed variably sized spots that localized to the interchromatin space (Fig. 1*G*). Using a small hairpin RNA (shRNA) to knock down the expression of endogenous PHF8, we observed that the number of P-LISA interaction hotspots was significantly reduced in cells expressing the shRNA (Fig. 1*H−I*), suggesting that the interaction detected by proximity ligation was specific. Immunostaining with the PHF8 antibody was performed to confirm that the interaction hotspots constituted a large majority of endogenous PHF8 puncta (Fig. 1*J*).

### PHF8 physically associates with TIP60

**Figure 2:**
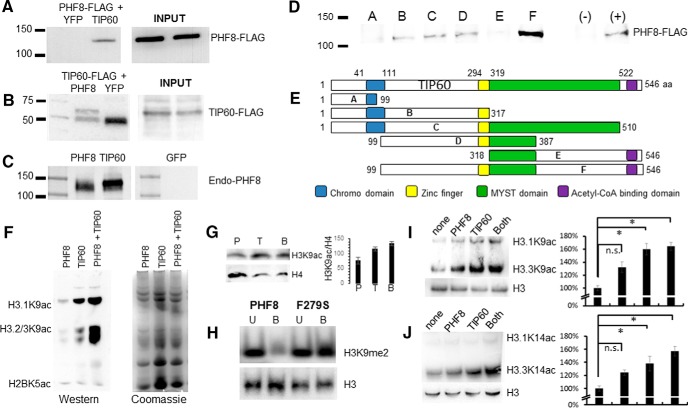
PHF8 and TIP60 physically associate to form a dual function chromatin-modifying complex. ***A***, Coimmunoprecipitation of PHF8 and TIP60 in HEK293T nuclear extracts, where TIP60−YFP was pulled down with anti-GFP antibody and PHF8−FLAG was detected with anti-FLAG by Western blotting. ***B***, Pulldown of PHF8−YFP showed that TIP60−FLAG was detected in the IP fraction but not in the YFP-only control lane. ***C***, Endogenous coimmunoprecipitation of PHF8 and TIP60 in DIV12 cortical neuronal nuclear extracts, showing that PHF8 is able to be pulled down by both the anti-PHF8 antibody and anti-TIP60 antibody, but not the anti-GFP antibody. ***D***, ***E***, Truncated constructs of TIP60 protein (A to F) containing the indicated TIP60 domain (E) were fused to YFP and then cotransfected with full-length PHF8−FLAG and immunoprecipitated with an anti-GFP antibody. Western analysis was performed to detect PHF8−FLAG in the immunoprecipitates using the anti-FLAG antibody. A negative control of YFP only is denoted by (-), whereas full-length TIP60 served as a positive control (+). ***F***, ***G***, Total histones from HEK293 cells overexpressing PHF8, TIP60, or both were separated on TAU gels (***F***) or conventional SDS-PAGE (***G***). Overexpression of TIP60 alone increases H3K9 acetylation in HEK293 cells for both the H3.1 and H3.3 isoforms, whereas acetylation of the non-TIP60 substrate H2BK5 was not affected. Coexpression of PHF8 and TIP60 increases H3.3K9 acetylation to even higher levels. ***H***, Chromatin immunoprecipitation using an antibody specific to H3K9me2, showing that overexpression of wild-type PHF8 but not the clinical mutant F279S (U, unbound or input levels of H3K9me2; B, bound or immunoprecipitated H3K9me2). ***I***, ***J***, ChIP assays of HEK293T cells transfected with PHF8, TIP60, or both analyzing histone tails positive for H3K4me3, the transcriptionally-activating histone mark that is known to be bound by PHF8, show that the increase in H3K9ac (***I***) and H3K14ac (***J***) is specific to histones carrying H3K4me3, and that this histone population was enriched in H3.3 (as shown by the more intense staining of this isoform on the TAU gel; asterisk). Western blot of the same lysates using an H3.3 antibody serves as loading control. The right panel shows bar graphs quantifying the increase in H3.3K9 and H3.3K14 acetylation, relative to the untransfected control (*n* = 3; *p* = 0.19 for PHF8 only, 0.04 for TIP60 only, 0.02 for PHF8+TIP60; asterisks indicates statistical significance: *p* < 0.05).

Despite a wealth of evidence suggesting that methylation and acetylation of histones are tightly linked, physical interactions between a histone demethylase and an acetyltransferase have not been well characterized. In light of our findings showing the colocalization of the demethylase PHF8 with the acetyltransferase TIP60 as well as their ability to recruit each other, we used a cellular system to study whether or not the two enzymes physically interact. HEK293 cells were cotransfected to >90% efficiency with TIP60−YFP and PHF8−FLAG, and their nuclear extracts were subjected to immunoprecipitation with the anti-GFP antibody to pull down TIP60-YFP. Control cells were transfected with YFP alone. After extensive rounds of washing, PHF8−FLAG remained in the IP fraction (Fig. 2*A*) of cells transfected with TIP60−YFP, but not of cells transfected with YFP alone. Conversely, when PHF8−YFP and TIP60−FLAG were cotransfected and coimmunoprecipitation was performed, only cells that contained PHF8−YFP immunoprecipitated TIP60−FLAG, whereas control cells transfected with YFP did not (Fig. 2*B*). In rat cortical neurons, pull-down of endogenous TIP60 with an anti-TIP60 antibody coimmunoprecipitated PHF8, whereas pull-down with anti-GFP did not (Fig. 2*C*). To analyze which domains of TIP60 physically associated with PHF8, we performed coimmunoprecipitation experiments where full-length PHF8−FLAG was coexpressed with deletion mutants of TIP60, which included the following TIP60 constructs: A (TIP60 chromo domain), B (N-terminus TIP60, including the zinc finger), C (N-terminus TIP60, excluding the acetyl-CoA binding domain), D (containing the zinc finger and part of its MYST domain), E (C-terminus TIP60, excluding the zinc finger), and F (C-terminus TIP60, excluding the chromo domain). Our results show that PHF8 associates most strongly with TIP60 amino acids 99-546, which contain the zinc finger or putative DNA-binding domain and the active acetyltransferase MYST domain (Fig. 2*D*, compare constructs D−F with E). In contrast, the N-terminal chromo domain, which recognizes and binds to the heterochromatin-associated H3K9me3, hindered its association with PHF8 (Fig. 2*D*, compare constructs C with D and F).

### PHF8 and TIP60 form a dual function complex that increases the acetylation of H3K4me3-bearing chromatin

The observed physical interaction between these two chromatin modifiers led us to explore whether the PHF8−TIP60 complex had any cooperative function in histone modification. We first investigated whether PHF8 and/or TIP60 had any effect on overall histone acetylation. Purified histones from HEK293 nuclear extracts obtained after transfection of PHF8, TIP60, or both were separated on TAU gels. We observed that overexpression of TIP60 increases histone acetylation of H3 at lysine 9 (H3K9), but when both PHF8 and TIP60 were overexpressed, the increase in H3K9 acetylation was notably larger (Fig. 2*F*). The same analysis was performed on total cellular histones separated on conventional SDS-PAGE gels, which shows that PHF8 and TIP60 increase total H3K9 acetylation, without affecting the loading control, total H4 (Fig. 2*G*).

The interaction between the active chromatin mark H3K4me3 and acetylation of H3K9 and H3K14 has been well documented (Rice and Allis, 2001), but the enzymes that cooperate to cause this cross-talk are not well understood. PHF8 is a reader of the transcriptionally associated mark H3K4me3, by virtue of its plant homeo domain (PHD) finger domain, while it functions to remove the repressive chromatin marks H3K9me2 and H3K9me1 (Kleine-Kohlbrecher et al., 2010; Yu et al., 2010). TIP60 is an acetyltransferase with predicted activity on many histone substrates including H3K9 and H3K14 (Kimura and Horikoshi, 1998). Following our observation that PHF8 associates with the acetyltransferase TIP60, we hypothesized that complete demethylation of H3K9me2 by PHF8 may be conducive to the acetylation of H3K9 and H3K14 in the same histone tail carrying the H3K4me3 mark. In order to test this hypothesis, we used ChIP to explore the possible functions of this complex on endogenous nucleosomes. We first validated the ability of our PHF8 construct to demethylate its substrate H3K9me2 using ChIP, and saw that while the wild-type enzyme was able to decrease H3K9me2, overexpression of the mutant version of PHF8−F279S, containing a single amino acid substitution that rendered it enzymatically inactive (Feng et al., 2010), did not result in a decrease in H3K9me2 (Fig. 2*H*). Pull-down was performed using an antibody directed against the PHF8-targeted mark H3K4me3, and the levels of acetylation at the H3K9 and H3K14 residues of these H3K4me3-positive histones were assessed by Western analysis. Consistent with its role as a histone acetyltransferase, upregulation of TIP60 expression was seen to increase acetylation of H3K9 and H3K14, but more importantly, this increase was specific to chromatin that is positive for H3K4me3. Interestingly, PHF8 overexpression by itself is also able to increase H3K9ac and H3K14ac levels. However, when both PHF8 and TIP60 are coexpressed, we observed an associated additive effect in the acetylation of H3 specifically in transcriptionally active H3K4me3-bearing histone tails (Fig. 2*I−J*). Taken together, both total histone gels and ChIP−TAU gels capturing specifically immunoprecipitated H3K4me3 show that there is proportionately higher H3K9 and H3K14 acetylation when both PHF8 and TIP60 are present, which lent further evidence that PHF8 and TIP60 may be acting cooperatively to increase H3 acetylation.

### PHF8 removes transcriptionally suppressive H3K9me2 and associates with transcriptionally active H3K9ac

**Figure 3:**
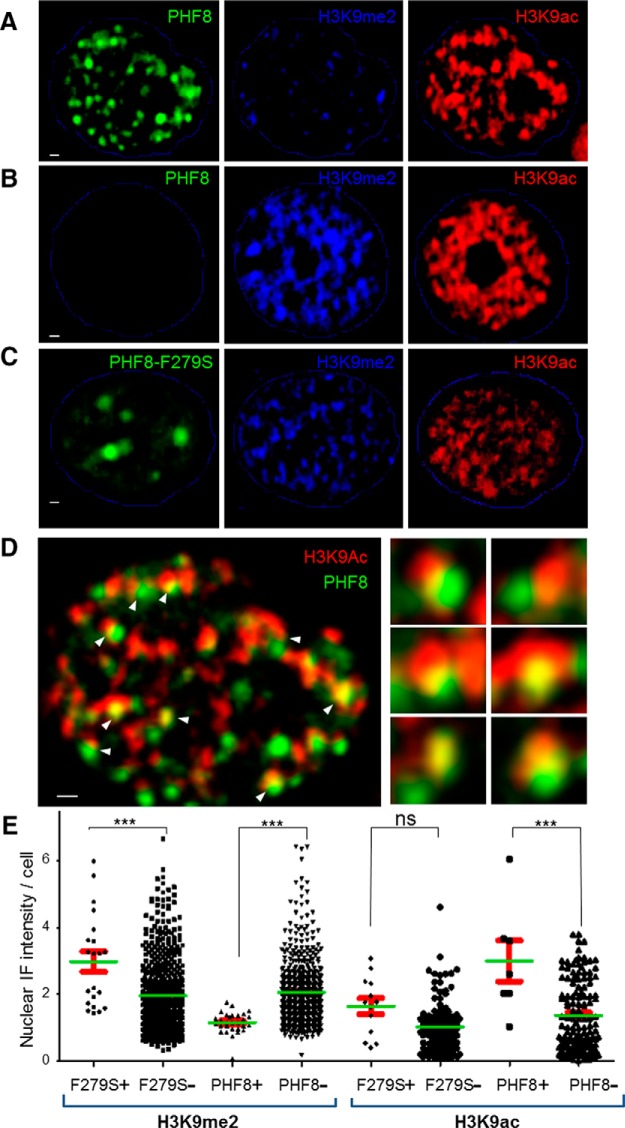
PHF8 removes the repressive histone mark H3K9me2 and associates with the activating histone mark H3K9ac. ***A***, A representative hippocampal neuronal nucleus outlined in blue, transfected with PHF8−CFP (pseudo-colored green), showing a marked decrease in the repressive chromatin mark H3K9me2 (pseudo-colored blue) in the nuclear domains occupied by PHF8, which is not seen when the neuron is untransfected (***B***) or when the mutant PHF8−F279S was transfected (***C***). ***D***, The same hippocampal nucleus depicted in ***A***, showing the association of PHF8 puncta with the histone acetylation mark H3K9ac (arrows point to regions of close apposition between PHF8 and H3K9ac, six of which are shown at higher magnification by the insets on the right; green+red = yellow). ***E***, Quantitation of the intensity of H3K9me2 staining in each nucleus (each symbol marks the H3K9me2 density of a single neuron), showing that PHF8-expressing neurons have significantly lower H3K9me2 density, whereas mutant PHF8 F279S-transfected neurons show the opposite effect (*** *p* < 0.0001; ns, not significant).

Previous studies have indicated that PHF8 may be a transcriptional coactivator, as it is able to demethylate the transcriptionally repressive histone mark H3K9me2 in various cellular models (Loenarz et al., 2010; Suganuma and Workman, 2010; Zhu et al., 2010). In the brain, the regulation of H3K9me2 has recently been found to be extremely important for learning and memory (Kramer et al., 2011). After confirming that PHF8 demethylates H3K9me2 in a cellular system (Fig. 2*H*), we sought to investigate whether PHF8 is able to reduce H3K9me2 levels in primary neurons. Indeed, PHF8 overexpression was sufficient to down-regulate H3K9me2 in hippocampal neurons, as seen through immunofluorescence (Fig. 3, compare *A*, *B*, and *C*). However, the clinical mutant PHF8−F279S displayed a different subcellular localization and function, localizing to fewer and slightly bigger foci in the nucleus and failing to down-regulate H3K9me2 (Fig. 3*E*). In view of the interaction between PHF8 and the acetyltransferase TIP60, we hypothesized that PHF8 may be regulating acetylation. We therefore screened a number of histone acetylation marks in neurons and found that the PHF8 puncta were seen to associate very closely with the acetylation mark H3K9ac, a transcriptionally permissive mark and known substrate of TIP60 (Kimura and Horikoshi, 1998) (Fig. 3*D*). These findings suggested that the PHF8−TIP60 complex may be responsible for mediating changes at a specific chromatin locus that was H3K9, the only residue known to be able to undergo either methylation or acetylation (Zlatanova and Leuba, 2004).

### PHF8 and TIP60 are activity-dependent and coregulate chromatin modification in response to neuronal activity

**Figure 4:**
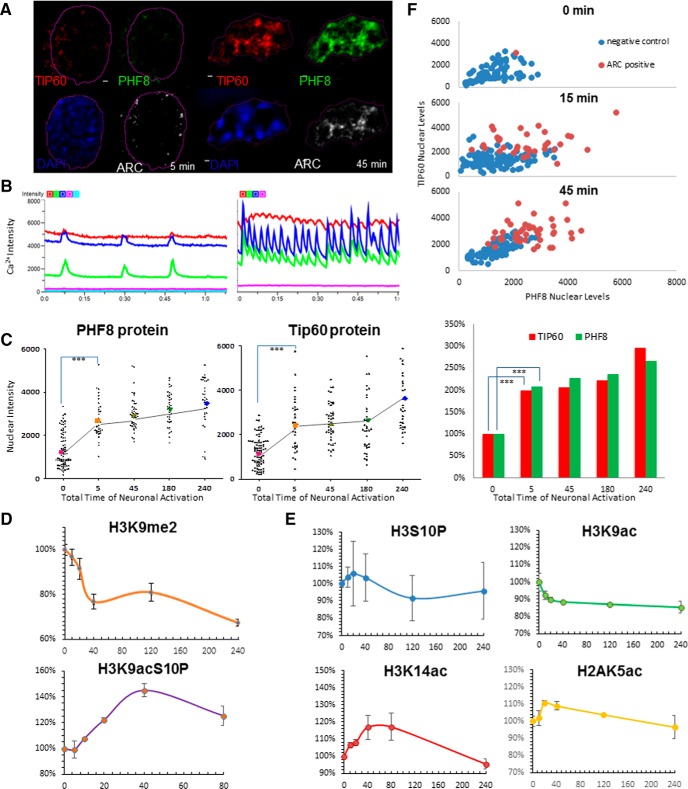
Neuronal activity reorganizes PHF8 and TIP60 in the nucleus and effectuate histone methylation and acetylation changes. ***A***, A representative image of a pair of hippocampal neuronal nuclei during the first 5 min of 4AP+Bic+Fors treatment and then at 45 min, showing the activity-dependent increase of PHF8 and TIP60 protein in the nucleus. ***B***, Neural network activity visualized by Ca^2+^ imaging (gCamp6 intensity over time), with each different-colored line representing individual neurons, before (left) and after (right) treatment with 4-AP+Bic. ***C***, Dot plot of nuclear levels of PHF8 and TIP60 (unpaired *t* test; *** *p* ≤ 0.0001; one-way ANOVA: *F* = 33.23, *R* ^2^ = 0.3693). Each symbol represents the intensity of PHF8 or TIP60 staining from a single neuronal nucleus, lines correspond to the mean and SEM of all neurons imaged at the indicated time points. ***D***, mRNA levels of PHF8 show a biphasic peak with time of chemLTP, whereas TIP60 shows an initial upregulation but a return to baseline within 45 min of sustained activity. ***E***, Time-courses of chromatin modification of neurons imaged using high-content screening (*n* = 500 − 1000/site, 6 sites/well, 96-well; ImageXpress Micro, Molecular Devices) show an activity-dependent decrease in the overall per-nucleus intensity of H3K9me2 staining in neural networks treated with chemLTP, which coincides with a robust increase in H3K9acS10P. ***F***, Graphs of nuclear PHF8 as a function of nuclear TIP60 levels at 0, 5, and 45 min of neural network activation in ARC-positive versus ARC-negative neurons, showing two identifiable distinct populations of neurons. The bar graph indicates levels of TIP60 (red) and PHF8 (green) as a function of time of synaptic activation (in minutes; y-axis). PHF8 and TIP60 are both highly induced within as early as 5 min of synaptic activation (*p* = 0.00001).

**Figure 5:**
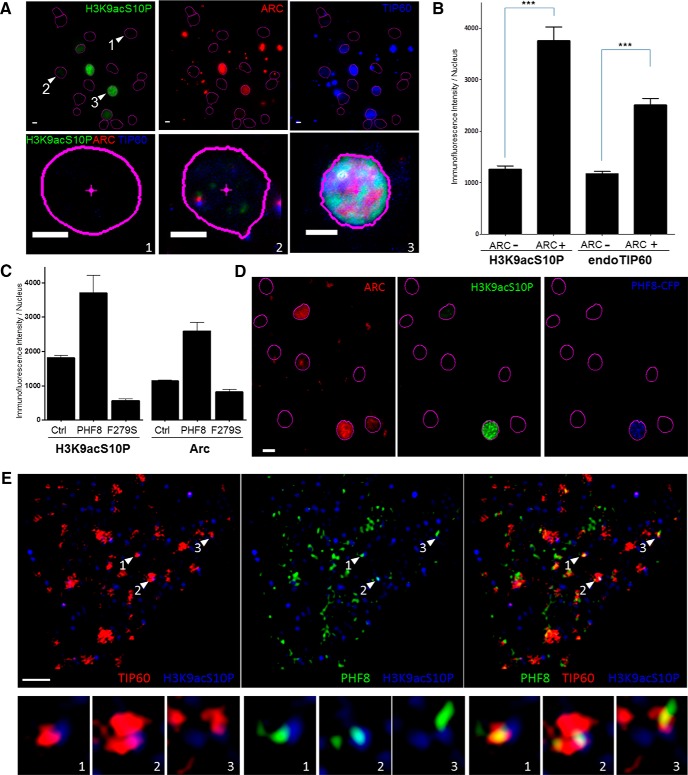
PHF8 and TIP60 modulate neuronal activity-induced histone acetylation at H3K9acS10P and activation of the *Arc* gene. ***A***, Representative microscopic field of hippocampal neurons after 1 h of network activation by chemLTP, showing a positive correlation between the expression of Arc (red) and Tip60 (blue) with the phosphoacetylation mark H3K9acS10P (green). The bottom panels show three different neurons that induced varying amounts of ARC protein. The neuron expressing the highest amount of ARC (3) also has high amounts of H3K9acS10P. ***B***, Quantification of 20 immunofluorescence-analyzed fields exemplified in ***A***, showing a statistically significant increase in H3K9acS10P as well as endogenous TIP60 in ARC-expressing neurons (*n* = 347 neurons; *** *p* = 0.00001). ***C***, Fusion constructs of PHF8 and its mutant F279S were individually expressed in hippocampal neurons and the next day the neuronal network was activated using ChemLTP (4AP+Bic+Fors). After 1 h of upregulated synaptic activity, the expression of PHF8, but not its mutant F279S, significantly increases histone acetylation at H3K9acS10P (*n* = 397 neurons; *p* = 0.00001). ***D***, A representative microscopic field of neuronal nuclei after 1 h of ChemLTP, with neuronal nuclei stained by DAPI outlined in magenta, showing the induction of ARC protein expression in a small subset of neurons, one of which had been transfected with PHF8−CFP (blue), and is high in H3K9acS10P (green). ***E***, A representative z-plane of a 3D SIM image of a neuronal nucleus after 1 h of chemLTP treatment, showing endogenous nuclear PHF8 puncta (green) and endogenous TIP60 (red) associating with the histone acetylation mark H3K9acS10P (blue). Arrows mark three selected regions, which are shown at higher magnification in the bottom panels, showing strong association between the PHF8−TIP60 complex and H3K9acS10P in the activated neuronal nucleus.

Although neuronal activity is known to induce epigenetic modification at the level of DNA methylation (Martinowich et al., 2003; Ma et al., 2009), the circumstances of activity-dependent chromatin modification is not well established. We attempted to investigate this by combining a chemical long-term potentiation (chemLTP) paradigm that stimulates network activity (Hardingham et al., 2001; Arnold et al., 2005) with a high-content imaging assay in 96-well format to examine global activity-dependent changes in histone methylation and acetylation as a function of time. We performed a detailed time course of neuronal activation at 5, 10, 20, 40, and 80 min intervals and beyond, and found that as early as 40 min after increasing synaptic activity, as visualized by the genetically-encoded calcium sensor GCaMP6 (Fig. 4*B*), expression of the activity-regulated gene *Arc* was induced (Fig. 4*A*). Activated neurons that successfully induce ARC protein had significantly higher nuclear levels of PHF8 and TIP60 (Fig. 4*C*,*F*, *p* < 0.0001), suggesting that both chromatin modifying enzymes are activity-dependent. In fact, a detailed time-course analysis of PHF8 and TIP60 nuclear levels in neurons that have undergone chemLTP treatment show increases in PHF8 nuclear levels, which is precisely mirrored by increases in TIP60 nuclear levels within 5 min of synaptic activity induction (Fig. 4*A*,*C*,*F*). In parallel with the increases in the nuclear levels of both PHF8 and TIP60, we found that neuronal networks activated with the chemical LTP paradigm showed a robust increase in the phosphoacetylation of H3K9acS10P, which was mirrored by a downregulation of the PHF8 substrate and transcriptionally repressive mark H3K9me2 (Fig. 4*D*), corroborating the data obtained through single-cell imaging (Fig. 5) and other studies of hippocampus-dependent memory formation (Chwang et al., 2007). Amongst various histone acetylation sites tested, including H3S10P, H3K9ac, H3K14ac, and H2AK5ac (Fig. 4*E*), we observed that H3K9acS10P was most responsive to synaptic activity, with low baseline levels and consistently reproducible induction within minutes of synaptic activation, suggesting that the very early phosphoacetylation of H3K9acS10P may constitute a highly specific chromatin signature of recently activated neurons.

### The PHF8−TIP60 complex modulates activity-induced H3K9S10P acetylation

Individual reports have shown that neurons are able to induce both H3S10 phosphorylation and histone acetylation in response to synaptic activity (Soriano et al., 2009; Wittmann et al., 2009), but the mechanisms by which this occurs and the possible functions of these modifications are not yet elucidated. Our observations indicate that the dual histone mark H3K9acS10P is a highly activity-dependent chromatin modification. In order to investigate whether PHF8 and TIP60 have an effect on the occurrence of H3K9acS10P, we performed high-resolution imaging of activated neuronal nuclei. Using immunofluorescence microscopy of neurons activated with chemLTP, we validated that ARC protein, which is known to be highly regulated by activity, was induced in only a subset of the neuronal population (Fig. 5*A*). In these neurons that were positive for ARC, nuclear levels of endogenous TIP60 highly paralleled the increase in H3K9acS10P (Fig. 5*B*), corroborating the results from the high-content analysis (Fig. 4).

**Figure 6:**
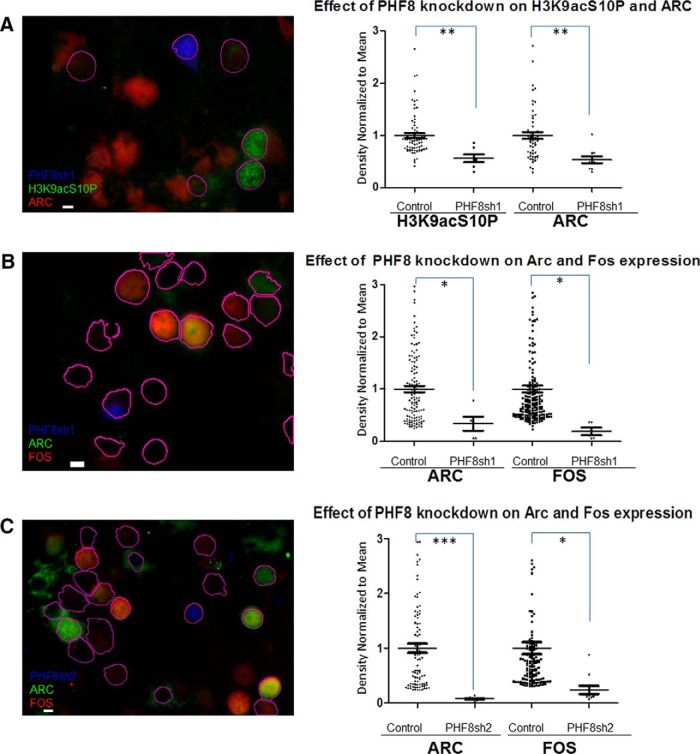
Knockdown of PHF8 impairs activity-dependent induction of H3K9acS10P and Arc and Fos expression. ***A***, Neurons transfected with PHF8 shRNA1 and subsequently treated with chemLTP activation for 3 h (DAPI-stained nuclei are outlined in magenta) were immunostained for H3K9acS10P and ARC. The right panel shows a corresponding quantification of the staining density (intensity/area/nucleus) normalized to the mean density for each condition, showing a significant decrease in H3K9acS10P induction as well as *Arc* gene expression (*p* = 0.0052). ***B***, ***C***, Representative microscopic fields showing neurons transfected with PHF8 shRNA1 (***B***) and PHF8 shRNA2 (***C***) and subsequently treated with chemLTP activation for 3 h (DAPI-stained nuclei are outlined in magenta), immunostained for products of immediate-early genes *Arc* and *Fos*. The right panel shows a corresponding quantification of the staining density (intensity/area/nucleus) normalized to the mean density for each condition, showing that shRNA knockdown by two individual PHF8 shRNAs succesfully inhibited *Arc* and *Fos* induction (***B***: *p* = 0.0301, 0.0408; ***C***: *p* = 0.0002, 0.0452; asterisks indicate the level of significance: * indicate *p* < 0.05; *** *p* < 0.001).

We then asked if PHF8 could affect this increase in the activity-dependent acetylation of H3K9acS10P. To answer this question, we transfected neurons with either wild-type PHF8 or the clinical mutant PHF8−F279S, and imaged them after chemLTP induction. Staining for both ARC and H3K9acS10P indicated that neurons with increased PHF8 have significantly higher H3K9acS10P and a higher probability of ARC protein expression (Fig. 5*C*,*D*). However, knockdown of PHF8 through transfection of two shRNAs significantly decreases H3K9acS10P (Fig. 6*A*). In order to more accurately delineate this interaction and to circumvent the problem that we could only visualize three proteins at a time immunohistochemically, we performed structured illumination microscopy, which revealed that the complex containing PHF8 and TIP60 directly associated with H3K9acS10P in the activated neuronal nucleus (Fig. 5*E*).

**Figure 7:**
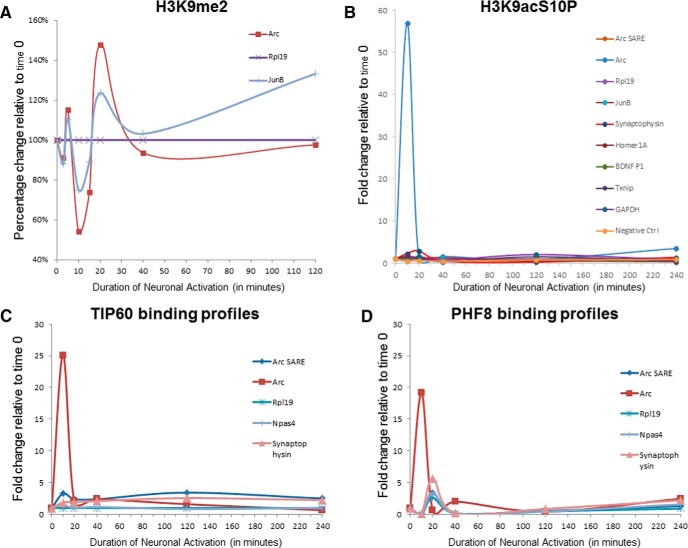
PHF8 and TIP60 are actively recruited to specific neuronal gene promoters. ***A***, ***B***, Within minutes of synaptic activation (*x*-axis: time of increased network activity, in minutes), time-course ChIP shows an early detectable decrease in the chromatin mark H3K9me2 at the *Arc* TSS (***A***), which is mirrored by a concomitant, highly transient increase in the levels of H3K9acS10P at the *Arc* TSS (***B***). This increase in H3K9acS10P was specific to the *Arc* promoter as analyses of *Rpl19*, *JunB*, *Synaptophysin*, *Homer1A*, *BDNF promoter 1*, *Txnip*, *Gapdh*, and *Fos* intergenic region (Negative Ctrl) did not show an activity-dependent increase. ***C***, ***D***, Time-course ChIP followed by qRT-PCR using primers against the transcriptional start site regions of the *Arc* gene, arc synaptic response element, ribosomal protein L19 (Rpl19), neuronal PAS domain protein 4 (Npas4), and synaptophysin. Both TIP60 (***C***) and PHF8 (***D***) are recruited to the Arc TSS within minutes of activation of the neuronal network, but not to the *Rpl19*, *Npas4*, or *Synaptophysin* transcriptional start sites.

Time-course chromatin immunoprecipitation of neurons activated by chemLTP showed that, while H3K9me2 levels decreased at the *Arc* TSS, H3K9acS10P was massively upregulated within 10 min of synaptic activity (Fig. 7*A*,*B*). Rapid, early recruitment of PHF8 and TIP60 to the *Arc* TSS (Fig. 7*C*,*D*), which coincided with a surge in H3K9acS10P phosphoacetylation at the same genomic locus (Fig. 7*B*), was not seen in other activity-dependent gene promoters and control genomic loci. Thus, taken together with the results obtained from high-content imaging, time-course ChIP data seems to suggest that the nuclear reorganization and active recruitment of PHF8 and TIP60 protein into gene promoters may be a possible mechanism by which neural activity causes changes in histone acetylation and methylation status that in turn influence the transcription of neuronal genes such as *Arc*.

### The PHF8−TIP60 interactome is rich in proteins involved in transcription and includes the neuronal splicing factor PSF

**Figure 8:**
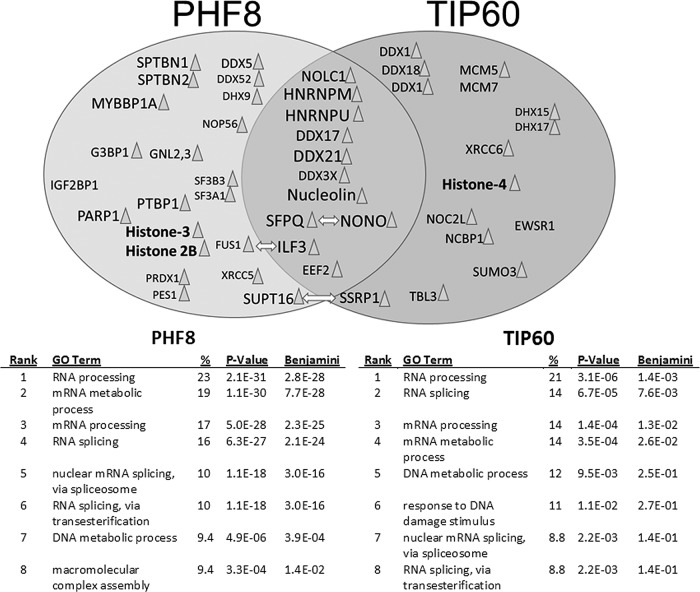
Common interacting partners between PHF8 and TIP60 function primarily in transcription and mRNA processing. Top, A Venn diagram showing several interacting partners of PHF8 and TIP60 as identified by immunoprecipitation followed by mass spectrometry. The overlapped region in the middle represents common partners that interact with PHF8 and TIP60, which include the splicing factor SFPQ (PSF) and its partner NONO, as well as several ATP-dependent RNA helicases, and the histone chaperone nucleolin. Proteins that have known acetylation sites are marked by a triangle (Choudhary et al., 2009). Arrows indicate known functional interactions between identified proteins. Font size indicates the percentage of the total protein that the identified MS/MS peptides covered (large font: >25% coverage; medium font: 5 − 25% coverage; small font: <5% coverage). Histone proteins identified in the IP−MS are in bold. Bottom, A listing of the top eight biological functions attributed to the proteins identified in the IP−MS of both PHF8 and TIP60 in order of abundance, as computed by the software DAVID (http://david.abcc.ncifcrf.gov/home.jsp), with the associated *p* value and Benjamini factor, showing that interactors of PHF8 and TIP60 are enriched in the functions of RNA processing, RNA splicing, and mRNA processing.

The identification of an activity-dependent chromatin-modifying complex may have wide-ranging implications in neuronal functions. We sought to investigate the possible functions of PHF8 and TIP60 by examining the protein partners they interact with. Both PHF8 and TIP60 have been implicated in various aspects of neuronal function and gene transcription (Kleine-Kohlbrecher et al., 2010; Tea and Luo, 2011), but a low-bias view of the PHF8−TIP60 interactome has not been established. We therefore performed immunoprecipitation followed by mass spectrometry of nuclear extracts from Hek293T cells transfected with PHF8−YFP or TIP60−YFP. The immunoprecipitates were run on a gel stained with Coomassie blue and bands were excised for mass spectrometric analysis. A large majority of the proteins that were found to immunoprecipitate with PHF8 and TIP60 had functions in transcription, splicing, and RNA processing (Fig. 8). Amongst the top interactors that were prominently represented by both the PHF8 and TIP60 IP-MS were the splicing factor SFPQ, also known as PSF, and its partner NONO (non-POU domain containing, octamer-binding), as well as several ATP-dependent RNA helicases including DDX17, DDX21, and DDX3X, which strongly suggested that PHF8 and TIP60 may be functioning in transcription-related processes.

### Endogenous PHF8, TIP60, and PSF are found within 30 nm of each other in the activated neuronal nucleus

**Figure 9:**
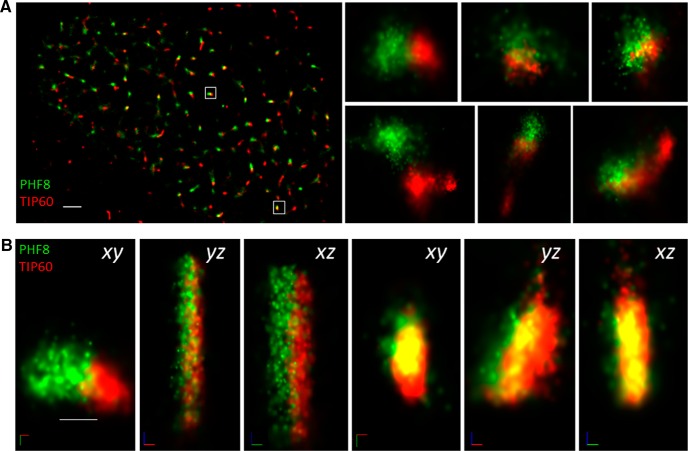
Endogenous TIP60 is located within 30 nm of PHF8 in the activated hippocampal neuronal nucleus. ***A***, A maximum intensity projection of a dual color 3D STORM image of a hippocampal neuronal nucleus that has undergone 1 h of chemLTP. The neuron has been labeled for endogenous TIP60 (red) and endogenous PHF8 (green), showing that the two molecules closely interact in various localized puncta in the nucleus. Scale bar, 1 μm. The insets on the right show six representative complexes at higher magnification (scale bar, 50 nm). ***B***, A highly magnified view of two endogenous PHF8−TIP60 complexes shown in the outlined area in ***A***. The insets on the right show three projections of the single-molecule interaction between PHF8 and TIP60 viewing down the x-, y-, and z-axes, demonstrating that the complexes formed between these two chromatin-modifying enzymes have well-defined spatial relationship. Each dot corresponds to the localization of a single molecule. Scale bars, 50 nm.

**Figure 10:**
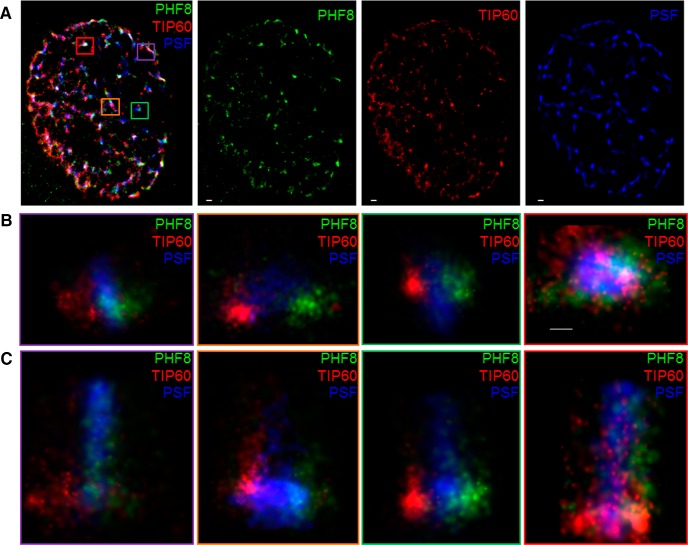
PHF8 and TIP60 form a tripartite complex with the splicing factor PSF and associates with newly transcribed nascent RNA. ***A***, A maximum intensity projection of a 3D STORM image of an activated hippocampal neuronal nucleus. Single-molecule imaging of endogenous PHF8 (green), endogenous TIP60 (red), and PTB-associated splicing factor (blue), with the corresponding single-channel views. Each dot corresponds to the localization of a single molecule. Scale bar, 500 nm. ***B***, ***C***, Four representative higher magnification views of the neuronal nucleus depicted in ***A***, showing that PSF (blue) forms a tailing structure within the interface between PHF8 (green) and TIP60 (red) viewed axially ***B*** or longitudinally ***C*** as a recognizable tripartite complex. Scale bar, 50 nm.

Although PHF8 and TIP60 are found to tightly colocalize when viewed using conventional immunofluorescence microscopy, it remains difficult to ascertain precisely how these chromatin-modifying enzymes are positioned relative to each other in the nucleus, as the resolution of a conventional light microscopy is limited to 250 nm, nearly 20× the diameter of a single nucleosome (11 nm). To overcome this hurdle, we employed 3D-STORM, which has a resolution limit of 20 nm, allowing us to directly observe endogenous PHF8 and TIP60 interactions at the single-molecule level. Indeed, as suggested by proximity ligation, single-molecule imaging by STORM shows that a large majority of PHF8 proteins were found to associate with TIP60 (Fig. 9). Within the interaction hotspots, PHF8 and TIP60 were found to colocalize in an interaction radius of 20 nm (Fig. 9*A*), which is less than the diameter of the 30 nm packed chromatin fiber, demonstrating for the first time the association of a histone demethylase and an acetyltransferase at level of a single nucleosome. As we had the capability to view these complexes three-dimensionally, we were able to observe that the single-molecule interaction between PHF8 and TIP60 did not occur in a random orientation, but rather had a specific spatial relationship (Fig. 9*B*; compare the projections in the xy, xz, and yz planes). Interestingly, we observed that molecules of PHF8 and TIP60 were found to form a linearly well defined interface, which prompted us to investigate what may be lying within. We investigated several candidate proteins garnered through the IP-MS screen (Fig. 8), and found that the splicing factor PSF was situated at this interface between PHF8 and TIP60: tricolor 3D-STORM imaging shows that PHF8, TIP60, and PSF form a well defined tripartite complex in the neuronal nucleus (Fig. 10; Movie 1).

Movie 1 Stochastic optical reconstruction microscopy of a tripartite complex formed by PHF8, TIP60, and the splicing factor PSF, rotated around the y-axis, showing the configuration of the epigenetic enzymes around a tail-like structure composed of the neuronal splicing factor PSF. 10.1523/ENEURO.0020-14.2015.video.1

## Discussion

Chromatin modification has a major role in the generation of complex behaviors, such as learning and memory (Alarcón et al., 2004; Peixoto and Abel, 2013). Paradigms of memory formation, such as contextual fear conditioning, induce changes in neuronal transcriptional programs through dramatic alterations of chromatin structure (Gupta et al., 2010; Bousiges et al., 2013), yet the mechanisms by which chromatin-modifying enzymes regulate gene expression in response to neuronal activity are still unclear (Roth and Sweatt, 2009; Vogel-Ciernia and Wood, 2014). Here, we show that the activity-dependent induction of memory consolidation genes is facilitated by a novel dual function chromatin-modifying complex. Specifically, demethylation of the transcriptionally suppressive H3K9me2 mark is linked to the acetylation of H3 through a specific interaction between the H3K9me2-specific X-linked mental retardation protein PHF8 and the Alzheimer’s disease-associated TIP60. Results presented in this study indicate that the PHF8−TIP60 complex may function as an epigenetic initiator of rapid *Arc* gene induction, likely by interacting with mRNA processing proteins such as the neuronal splicing factor PSF and by upregulating H3K9acS10P, a chromatin modification that highly favors transcriptional elongation at the *Arc* transcriptional start site.

### A chromatin-modifiying complex that regulates histone methylation and acetylation in response to synaptic activity

Neuronal cells respond to environmental cues by altering chromatin signatures that affect gene transcription (Crosio et al., 2003). Whether or not specific histone marks are involved in transcriptional activation is the subject of intense research. Histone acetylation is thought to be a transcriptionally permissive modification that is important in memory formation (Peixoto and Abel, 2013), while histone methylation may be activating or repressive (Jarome and Lubin, 2013). It has been known for a long time that both modifications are related to transcription (Allfrey et al., 1964) and recent evidence suggests that histone acetylation and methylation may be regulated cooperatively to activate transcription (Latham and Dent, 2007). For instance, histone acetylation correlates strongly with methylation in the context of gene induction (Zhang et al., 2004; Nightingale et al., 2007). In hippocampal neurons, electroconvulsive seizures induce changes in transcription by altering chromatin acetylation and methylation in a locus-specific manner (Tsankova et al., 2004). Nevertheless, little is known about how the enzymes responsible for acetylation and methylation cooperate and which chromatin modifications, if any, are required for neuronal gene transcription.

Studies done in *Drosophila* and mammals support the evolutionarily conserved role of a particular histone methylation mark, H3K9me2, in learning and memory (Gupta-Agarwal et al., 2014). PHF8 is a H3K9me2-specific histone demethylase that is clinically found to be mutated in a severe form of X-linked mental retardation (Laumonnier et al., 2005). PHF8 possesses a PHD finger domain targeting it to transcriptional start sites, and a Jumonji catalytic domain that is capable of removing the transcriptionally repressive mark H3K9me2/1 and H4K20me1 (Qi et al., 2010). The role of PHF8 mutations in causing mental retardation has been attributed to the inability of mutant PHF8 to activate ribosomal DNA transcription (Feng et al., 2010). Other studies suggest that PHF8 is a positive regulator of mRNA transcription, as it physically associates with RNA polymerase II along with transcription factors such as c-Myc and E2F (Asensio-Juan et al., 2012). Like PHF8, TIP60 has been found to be recruited to chromatin by c-Myc and E2F, where it has a function in transcriptional activation of both ribosomal and messenger RNA (Sapountzi et al., 2006). Although it has an emerging neurological phenotype (Lorbeck et al., 2011) and has been implicated in the pathogenesis of Alzheimer’s disease (Cao and Südhof, 2001), TIP60 has no currently known function in activity-dependent gene induction.

We report evidence that both PHF8 and TIP60 are located in the interchromatin space where transcription is thought to occur (Tycon et al., 2014). PHF8 shows a unique subnuclear distribution constituting a large number of small puncta, whose localization overlapped robustly with that of the acetyltransferase TIP60 (Fig. 1*D*). Furthermore, exogenous PHF8 is seen to recruit endogenous TIP60 and vice versa, while proximity ligation and coimmunoprecipitation experiments both demonstrate that PHF8 interacts with TIP60. This physical interaction and the observation that overexpression of a demethylase increases histone acetylation prompted us to ask whether PHF8 and TIP60 may be cooperatively modifying chromatin. Cross-talk between histone acetylation and methylation has been noted to be biologically significant (Kennedy et al., 2013), yet the interaction between demethylation and acetylation, along with the identity of the enzymes responsible for these changes are still unknown. The data presented here shows that PHF8 and TIP60 increase H3 acetylation predominantly on transcriptionally permissive H3K4me3-bearing chromatin, suggesting that the increase in acetylation may be specific to transcriptionally active genomic locations.

Given the identification of a complex that is capable of cooperatively modifying chromatin, we now address the issue of which specific histone marks may be regulated in response to neuronal activity. We observed that PHF8, but not the clinical mutant PHF8−F279S, is able to specifically downregulate the transcriptionally suppressive histone mark H3K9me2, consistent with its role as a transcriptional coactivator. However, besides removing H3K9me2, PHF8 tightly associates with H3K9ac, an important histone mark that is associated with paused RNA polymerase II (Margaritis and Holstege, 2008). This unexpected association was the first clue that demethylation and acetylation may be linked in neurons, specifically at the H3K9 epigenetic locus. Besides being implicated in evolutionarily conserved roles of learning and memory, the H3K9 locus is peculiar in that it can be acetylated or methylated, often with opposing biological consequences. In order to examine which histone marks are activity-regulated, we used a high-content screening platform to observe epigenetic changes at the network level, where we found that a treatment paradigm consisting of 4-aminopyridine, bicuculline, and forskolin, which has been shown to increase synaptic activity and cause LTP (Otmakhov et al., 2004), increases nuclear levels of PHF8 and TIP60 protein specifically in neurons that successfully induce the immediate-early gene *Arc* (Fig. 4*A−C*). Consistent with the increase in PHF8, we found that activity transiently downregulated the PHF8 substrate H3K9me2 (Fig. 4*D*). To our surprise, while immunostaining with the H3K9ac and H3K14ac antibodies did not show a robust regulation with regard to activity (Fig. 4*E*), we detected a highly specific activity-regulated increase in the dual histone mark H3K9acS10P (Fig. 4*D*), suggesting that a highly specific chromatin switch may exist in the bivalent H3K9 residue, but only in the context of S10 phosphorylation. Our findings thus characterize H3K9acS10P as an epigenetic chromatin signature that is faithfully produced by synaptic activity. The importance of the dual-mark H3K9acS10P is corroborated by previous reports that show that contextual fear conditioning and novel environment exposure induced changes in H3S10 phosphorylation, H3 acetylation, and H3K9me2 demethylation (Levenson et al., 2004; Chwang et al., 2006; Gupta et al., 2010; Gupta-Agarwal et al., 2014). A direct mechanism of combinatorial histone modification proposed by our study is that demethylation of the H3K9 residue by PHF8 in turn allows for its acetylation by TIP60 in the dual function complex, which may happen concurrently with or following the phosphorylation of the adjacent H3S10 (Duan et al., 2008). Further research is needed to optimally dissect the pathways that converge on H3S10 phosphorylation and the role that PHF8−TIP60 may play in this process.

### Regulation of neuronal gene transcription by H3K9acS10P, a chromatin modification specific for transcriptional elongation

What could be the function of this neuronal activity-regulated chromatin modification complex? Clinically, mutations in PHF8 protein that render it enzymatically inactive cause severe cognitive deficits (Laumonnier et al., 2005; Loenarz et al., 2010). While no known clinical mutation in TIP60 has yet been reported, nervous system-specific loss of TIP60 acetyltransferase activity dramatically worsens the Alzheimer’s disease phenotype in *Drosophila* (Pirooznia et al., 2012; Johnson et al., 2013; Xu et al., 2014). Furthermore, recent research shows that both PHF8 and TIP60 have important roles in transcriptional elongation (Halkidou et al., 2004; Wang et al., 2009; Mahajan and Stanley, 2014). Taken together, this evidence strongly point toward a role for PHF8 and TIP60 in transcriptional activation.

Given our current findings, we postulate that the PHF8−TIP60 complex may be functioning to increase the expression of neuronal genes such as *Arc* through the enzymatic modulation of H3K9acS10P, which is a highly important chromatin modification that mediates transcriptional elongation (Macdonald et al., 2005; Winter et al., 2008; Zippo et al., 2009; Karam et al., 2010; Li et al., 2013). Three lines of reasoning led to this conclusion. First, PHF8 and TIP60 nuclear levels paralleled the activity-dependent increase of H3K9acS10P and the surge in the expression of *Arc*, which became upregulated from extremely low baseline levels. Second, overexpression of PHF8 significantly enhanced the formation of H3K9acS10P and ARC protein, whereas the X-linked mental retardation mutant PHF8−F279S failed to produce the same effect, indicating that the demethylase activity of PHF8 is critical. Consistent with this view, inhibition of PHF8 gene expression using two different shRNAs significantly decreased H3K9acS10P induction. Third, using high-resolution structured illumination microscopy, we directly show that both PHF8 and TIP60 are tightly bound to H3K9acS10P. In summary, neuronal PHF8 and TIP60 may influence gene transcription by acting as activity-dependent writers of H3K9ac, which is known to be upregulated in novel environment exposure (Bousiges et al., 2013) and fear conditioning (Peleg et al., 2010), but only in the context of H3S10 phosphorylation, which has previously been shown to be critical for associative memory (Levenson and Sweatt, 2005).

The link between H3K9acS10P and transcription has been made in light of recent evidence showing that H3K9acS10P is required for transcriptional elongation through recruitment of the double bromodomain enzyme BRD4 and the elongation factor pTEF-b (Ong and Corces, 2011). Here, we show that in neurons, PHF8, TIP60, and H3K9acS10P were found to be specifically enriched in the *Arc* TSS in an activity-dependent manner (Fig. 7*B−D*). An intriguing possibility raised by this study is that upregulation of H3K9acS10P by PHF8 and TIP60 may serve as the initial impetus that transduces synaptic activity to the nucleus to drive the rapid transcription of *Arc*, which is poised for near-instantaneous transcription through promoter-proximal Pol II stalling (Saha et al., 2011). It is worth noting that the highly rapid transcriptional activation through release of Pol II into the elongation state is a conserved and well characterized mechanism of gene induction (Hargreaves et al., 2009). By allowing for the locus-specific formation of H3K9acS10P, which may serve as a platform for binding of elongation factors, the PHF8−TIP60 complex may be tipping the scale in favor of transcriptional elongation in response to synaptic activity.

Further evidence implicating this complex in the active transcriptional process is our finding that an overwhelming majority of the binding partners of PHF8 and TIP60 consisted of proteins involved in mRNA splicing and processing (Fig. 8). One highly represented protein in the PHF8−TIP60 interactome is the RNA polymerase II-associated splicing factor PSF (Emili et al., 2002; Rosonina et al., 2005), which is reported to play a key role in neuronal development (Lowery et al., 2007), alternative splicing (Kim et al., 2011), and is found to be dysregulated in neurodegenerative diseases (Ke et al., 2012). Although the interaction between PHF8 and PSF may be partly explained by the affinity of these proteins to the C-terminal domain of RNA polymerase II (Fortschegger et al., 2010), the highly structured molecular interaction between PHF8, TIP60, and the splicing factor PSF (Fig. 10) points towards a transcriptional role for the PHF8−TIP60 complex and supports the idea that pre-mRNA processing may be occuring cotranscriptionally (Ameur et al., 2011; Lee and Tarn, 2013; Bentley, 2014) .

In an attempt to directly observe this neuronal activity-regulated chromatin-modifying complex, we characterized the PHF8−TIP60 interaction in the neuronal nucleus at the single-molecule level using the single-molecule imaging technique 3D-STORM (Rust et al., 2006; Huang et al., 2008). Analysis at super-resolution reveals that interactions that are reportedly overlapping at the resolution of conventional light microscopy may be more complex than they seem, as we see PHF8 and TIP60 molecules not colocalizing perfectly but rather tightly associating with each other in an interaction radius of less than 20 nm, which comes increasingly close to the resolution needed to observe the “beads on a string” structure of nucleosomes on which general transcription is thought to occur (Smolle and Venkatesh, 2014). It was only upon three-dimensional rendering, however, that we could see these chromatin-modifying enzymes interacting in a well defined spatial orientation with a clear interphase (Fig. 9*B*). Intriguingly, consistent with IP-MS data, our work using tricolor 3D-STORM situates the splicing factor PSF in the middle of this interface between PHF8 and TIP60 (Fig. 10), offering a glimpse into the mechanism of cotranscriptional splicing in activated neuronal nuclei.

Could a chromatin mark such as H3K9acS10P serve as a regulator of memory formation processes through the modulation of transcription? If so, can the ability of neurons to change chromatin structure be altered enzymatically to facilitate or inhibit activity-dependent gene induction? The findings summarized in this report point to a mechanism by which synaptic activity may be transduced into the nucleus as an early epigenetic event that mediates downstream processes such as transcription. PHF8 is unique in that it has a PHD domain that is specific for transcriptionally active H3K4me3 coupled to demethylase activity against transcriptionally suppressive H3K9me2/1 and H4K20me (Vermeulen et al., 2010), making it well suited to be a transcriptional activator (Perner and Chung, 2013). Transcription elongation, however, requires extensive histone acetylation of residues such as H3K9 and H4K16, which serve as nucleosomal binding sites for BRD-4 to increase RNA Pol II processivity (Zippo et al., 2009). By associating with the histone acetyltransferase TIP60 and activating histone acetylation in a highly targeted manner, PHF8 may indeed serve as the link between transcription initiation and elongation. A possible mechanism that may therefore be proposed is rapid phosphoacetylation of H3K9acS10P by activity-dependent PHF8 and TIP60 may promote the escape of stalled RNA polymerase II and thereby transcription of immediate-early genes. A direct consequence from such a proposal is that abrogation of PHF8 function would prove to be detrimental towards learning-induced neuronal H3K9acS10P formation and the resulting neuroplasticity-related gene expression programs. Hence, the findings described here at least partly explains the limited capability to consolidate memories seen in patients who lack functional PHF8, and instigates further research into the possibility of altering activity-dependent gene transcription by modulation of epigenetic enzymes.

In summary, this work supports the idea that a neuronal dual-function chromatin-modifying complex containing PHF8 and TIP60 may serve as an epigenetic gateway to memory formation processes by regulating H3K9acS10P, a learning-induced, activity-dependent chromatin mark that enables *de novo* activity-dependent gene transcription. Future research into the modulation of epigenetic enzymes such as these may have potential applications in the development of novel therapeutics for disorders of learning and memory.

## Figures and Tables

**Figure:**
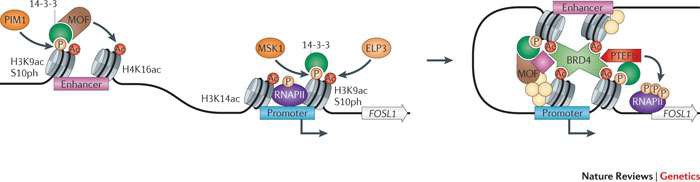
Serum stimulation induces proviral integration site 1 (PIM1)-mediated phosphorylation (P) of pre-acetylated (Ac) H3K9S10 (serine 10 of histone H3 lysine 9) at the FOS-like antigen 1 (*FOSL1*) enhancer. PIM1 is a proto-oncogene-encoded serine/threonine protein kinase. Binding of 14-3-3 to nucleosomes that carry histone H3 acetylation at lysine 9 and phosphorylation at serine 10 (H3K9acS10ph) recruits MOF, a histone H4 lysine 16-specific acetyltransferase, which in turn acetylates H4K16 (H4K16ac). In *Drosophila melanogaster*, elongator complex protein 3 (ELP3) and JIL1 (a serine/threonine kinase that is related to nuclear mitogen- and stress-activated protein kinase 1 (MSK1 in humans)) respectively acetylate and phosphorylate H3K9S10 at the promoter. **The modified nucleosomes act as a platform for the sequential recruitment of bromodomain-containing 4 (BRD4) and positive transcription elongation factor b (PTEFb).** PTEFb then facilitates the release of the promoter-proximal paused RNA polymerase II (RNAPII). The type of Mediator (shown by a purple diamond) and cofactors (shown by yellow circles) involved remains to be determined. *Nature Reviews Genetics* **12**, 283-293 (April 2011)

## Synthesis

The decision was a result of the Reviewing Editor Michael Sofroniew and the peer reviewers coming together and discussing their recommendations until a consensus was reached. A fact-based synthesis statement explaining their decision and outlining what is needed to prepare a revision is listed below.

The reviewers and editors agree that there is good consensus among the review comments and requests. The manuscript has strengths but requires major revision and re-review. Various specific concerns must be addressed, and the manuscript needs streamlining, clarification and focus to make it more accessible.

### Reviewer #1

In the manuscript entitled "A dual function chromatin-modifying complex regulates neuronal activity-dependent gene transcription," the authors present a beautiful, albeit dense, data set indicating the following: 1) The PHD finger protein PHF8 and the HAT TIP60 co-localize with one another in non-heterochromatic regions of cultured hippocampal nuclei; 2) PHF8 and TIP60 physically interact in HEK293T cells and associate in complex with H3.3K9 and K14ac; 3) PHF8 antagonizes H3K9me2 to promote H3K9ac in neurons; 4) PHF8 and TIP60 re-organize together in the nucleus of neurons following chemical LTP; 5) This complex modulates H3K9S10P to promote Arc expression in response to neuronal activity; 6) PHF8 and TIP60 form a tripartite complex with PSF (a splicing factor) to associate with nascent transcribed RNA in activated neurons; and 7) PHF8 and TIP60 are both induced in response to novel environment exposure in adult mouse hippocampus, with H3K9acS10P being induced in PHF8/TIP60 positive nuclei. I think that the manuscript is well written, the data are convincing and that this manuscript is appropriate for publication in eNeuro. I do, however, have concerns that need to be considered.

1) The biochemical analyses presented in Figure 3 indicating 'direct' binding interactions between PHF8 and TIP60 were performed in HEK293T cells, whereas all other analyses were performed in neurons. Although I understand that primary neurons present challenges for such analyses, it seems inappropriate to extrapolate information gained from HEK293T cells to neurons. In particular, to make claims about complex associations with H3.1 vs. H3.3 PTMs seems ill conceived given that histone variant distributions and PTM enrichment patterns likely differ dramatically between post-replicative neurons and rapidly proliferating HEK293T cells. My suggestion would be to repeat some of these IPs in neurons and minimize discussions about associations with H3.1 vs. H3.3 unless further data is presented to support these claims.
2) It is unclear to me what the authors believe the role of H3K9acS10P vs. H3K9ac alone is in relation to the binding interactions presented. The data are clear that PHF8 antagonizes H3K9me2 and that interactions with TIP60 promote acetylation, but why do the authors choose to focus so much attention on S10P. In most circumstances, the addition of adjacent S10P to either K9me1/2/3 or K9ac kicks off binding complexes normally associated with K9 events alone. If the authors choose to maintain a focus on this dual modification, then they need to perform subsequent experiments to convince me that their abilities to bind H3 are maintained both in the absence and presence of S10P. Given that this might require a substantial effort (e.g., modified histone peptide IPs in vitro, etc.), perhaps they should refocus their attention to the acetylation mark itself. It is my opinion that focusing on H3K9acS10P detracts from the story, as the authors chose not to address the function of this combinatorial readout directly.
3) Although the authors focus exclusively on the Arc gene, the data of which are convincing, I wonder why a more global genome-wide approach wasn't taken? To truly demonstrate that this complex globally controls activity-dependent transcription, I would need to see ChIP-seq experiments performed. Alternatively, the authors might consider significantly dampening their claims about the global role of this complex in transcription.
4) I am very glad that the authors present in vivo data, however, without presenting any biochemistry in the adult brain showing that PHF8 and TIP60 do indeed display increased binding after novel environment exposure, then I feel that the inclusion of these data are unnecessary.
5) Lastly, it is my opinion that this manuscript is entirely too dense and needs to be streamlined significantly. As I said, the data presented are very nice, but inclusion of 13 figures to make their points seems excessive. I feel that a non-chromatin specialist would simply be lost.

### Reviewer #2

In "A dual function chromatin-modifying complex regulates neuronal activity-dependent gene transcription" the authors demonstrate for the first time the interaction of the demethylase PHF8 and the acetyltransferase TIP60 to regulate gene induction by neuronal activity. This interaction, which is consistently documented throughout the paper using diverse approaches, may have important consequences in regulating cognitive processes and may be a relevant target in neuropathologies with cognitive decline, such as intelectual disabilities and Alzheimer's disease. However, examination of a learning paradigm has not been within the scope of the present work. Several concerns should be addressed to make the conclusions stronger and to facilitate the reading: the high amount of figures and the variety of experiments requires an additional effort in the presentation of the data for a better comprehension.

1. The title of the manuscript suggest that this is a general mechanism, but so far only one activity-dependent gene has been examined in detail (Arc) and another one does not show the same behavior (Npas4 in Figure 8).
2. To some extent there is some inconsistency in presenting data in a balanced way for PHF8 and TIP60: for example, only a deletional dissection of TIP60 structure is shown in Figure 3, the concomitant quantification of H3K9ac is missing in Figure 4, in Figure 14 TIP60 induction is not shown whereas an overlay/correlation of PHF8 with Arc is lacking in later panels.
3. It is concluded that TIP60 terminal chromo binding hinders association with PHF8 but construct E does not contain the N-terminus portion. Moreover, to conclude the effect of construct F it is important to know whether the presented results are representative of a series of duplicates.
4. The band intensities in Figure 3f are too uneven to make a conclusion of the experiment without a quantification.
5. It is concluded from results in Figure 3h/i that the effect of transfecting both proteins is synergistic on acetylation of histone H3 but the difference between TIP60 and TIP60+PHF8 lanes is not obvious. In any case, as it is, it is relevant in the context of the present study to demonstrate a physical binding between between H3K4me3 and H3K9ac that is enhanced in overexpressing conditions.
6. Check x-axis labels in Figure 4e: PHF8- is without overexpression of PHF8 but showes the lowest levels of dimethylation, the opposite of panel a and b; it is not obvious what it means F279S- (wild-type?).
7. The authors indicate that "various acetylation sites tested, we observed that H3K9acS10p was the most responsive to synaptic activity". It is important to show the tested marks, and more considering that the responsiveness of H3K9acS10p may be by the sole modification of Serine 10 as the antibody may recognizes either the acetylation or phosphorylation. Because the basal signal for the immunohistochemistry (Figure 14) is neglectable, it is reasonable to think that the antibody only recognizes the phosphorylation change, which it is virtually absent in basal state, therefore compromising the link with the acetylation and the enzymatic function of PHF8-TIP60.
8. Figure 8 contains a highly important message (the binding of TIP60 and PHF8 at the Arc promoter) that is worthy to indicate in the main text. Is it possible to add the profile of H3K9me2 in panel a?
9. The introduction of the mass spectrometry in HEK293 cells breaks the flow of the manuscript in its present form. Apart from providing a testable candidate in Figure 12, it is not obvious the main message of this experiment: is the overlap significant? Consider to present earlier this piece of information, before the work with neuronal cultures. In addition, nucleolin appears as a common interacting partner but Figure 2 indicates lack of colocalization of PHF8-nucleolin.
10. It is intriguing that no signal is observed in the basal state for either PHF8 and TIP60 in Figures 13 and 14 as they can be expressed in brain. A quantitative RT-PCR may solve this issue.

### Minor points:

1. In the Introduction it is said that "stimulus-dependent rapid gene induction... is heavily dependent on dynamic chromatin modification". Be more precise, as certain aspects of epigenetic-based regulation are more complex as originally proposed in cognitive and activity-dependent gene expression (see the review by Lopez-Atalaya and Barco in TiG). In addition, the provided references focus on the histone-code hypothesis rather than review the evidences supporting the present work; more recent reviews may be more appropriate (for example, Sweatt's commentary in Neuron).
2. Explain better the P-LISA technique, as readers may be more familiar with FRET-based techniques. This approach is highly relevant to demonstrate the interaction PHF8-TIP60 and it is not even mentioned in the Methods section.
3. Show the DAPI channel alone in Figure 1c to verify that do not significantly overlap with the labeling of PHF8 and S5P.
4. It is said that the mutation F279S of PHF8 is clinically relevant. Specify a reference. Is it a dominant negative? An answer will clarify the results of Figure 1i.
5. In Figure 3g a loading control is missing.
6. The low intensity in the staining of both modified histones in Figure 4c may be misleading.
7. Add axis labels in Figure 5.
8. References to Figure 2e is not correct in the section "PHF8 and TIP60 are members of a dual function complex, etc" and to Figure 7b-c when talking about H3K9acP10s reduction.
9. Consider to merge Figure 13 and 14 (or to remove Figure 13).

## Author Response

The reviewers and editors agree that there is good consensus among the review comments and requests. The manuscript has strengths but requires major revision and re-review. Various specific concerns must be addressed, and the manuscript needs streamlining, clarification and focus to make it more accessible.

### Reviewer #1

In the manuscript entitled "A dual function chromatin-modifying complex regulates neuronal activity-dependent gene transcription," the authors present a beautiful, albeit dense, data set indicating the following: 1) The PHD finger protein PHF8 and the HAT TIP60 co-localize with one another in non-heterochromatic regions of cultured hippocampal nuclei; 2) PHF8 and TIP60 physically interact in HEK293T cells and associate in complex with H3.3K9 and K14ac; 3) PHF8 antagonizes H3K9me2 to promote H3K9ac in neurons; 4) PHF8 and TIP60 re-organize together in the nucleus of neurons following chemical LTP; 5) This complex modulates H3K9S10P to promote Arc expression in response to neuronal activity; 6) PHF8 and TIP60 form a tripartite complex with PSF (a splicing factor) to associate with nascent transcribed RNA in activated neurons; and 7) PHF8 and TIP60 are both induced in response to novel environment exposure in adult mouse hippocampus, with H3K9acS10P being induced in PHF8/TIP60 positive nuclei. I think that the manuscript is well written, the data are convincing and that this manuscript is appropriate for publication in eNeuro. I do, however, have concerns that need to be considered.

1) The biochemical analyses presented in Figure 3 indicating 'direct' binding interactions between PHF8 and TIP60 were performed in HEK293T cells, whereas all other analyses were performed in neurons. Although I understand that primary neurons present challenges for such analyses, it seems inappropriate to extrapolate information gained from HEK293T cells to neurons. In particular, to make claims about complex associations with H3.1 vs. H3.3 PTMs seems ill conceived given that histone variant distributions and PTM enrichment patterns likely differ dramatically between post-replicative neurons and rapidly proliferating HEK293T cells. My suggestion would be to repeat some of these IPs in neurons and minimize discussions about associations with H3.1 vs. H3.3 unless further data is presented to support these claims.
  Sentences referring to “direct” binding have been rephrased appropriately. We agree with Reviewer #1 that the distributions and PTM enrichment patterns may be different between post-mitotic neurons and rapidly proliferating HEK293T cells, necessitating a more careful interpretation of the data – hence, all sentences with these interpretations have been removed. What we can interpret from the results is that the effect of PHF8 and TIP60 overexpression in HEK293T cells is an increase in H3K9 acetylation globally in both the H3.1 and H3.3 isoforms (Fig 3f.). However, it was only after pulldown with an antibody against H3K4me3 that we found that H3K4me3-positive histones were in fact predominantly H3.3 (compare the top bands from the bands with the red asterisks in Fig. 3h and Fig. 3i) at least in HEK293T cells and it is in these populations that we saw the effect of increased H3K9 and H3K14 acetylation due to PHF8 and TIP60. These experiments were driven by the fact that PHF8 is a known binder of H3K4me3 which targets it to transcriptionally active regions and tightly modulates its demethylase activity against H3K9 (please see in-text reference Fortschegger et al. 2010). Due to reasons of poor transfection efficiency, we were not able to do these experiments in cortical neurons; therefore, we have changed the phrasing in the text appropriately.
2) It is unclear to me what the authors believe the role of H3K9acS10P vs. H3K9ac alone is in relation to the binding interactions presented. The data are clear that PHF8 antagonizes H3K9me2 and that interactions with TIP60 promote acetylation, but why do the authors choose to focus so much attention on S10P. In most circumstances, the addition of adjacent S10P to either K9me1/2/3 or K9ac kicks off binding complexes normally associated with K9 events alone. If the authors choose to maintain a focus on this dual modification, then they need to perform subsequent experiments to convince me that their abilities to bind H3 are maintained both in the absence and presence of S10P. Given that this might require a substantial effort (e.g., modified histone peptide IPs in vitro, etc.), perhaps they should refocus their attention to the acetylation mark itself. It is my opinion that focusing on H3K9acS10P detracts from the story, as the authors chose not to address the function of this combinatorial readout directly.
  We would like to thank the Reviewer for an extremely insightful and highly relevant critique of our work. Indeed, we are focusing on the active **de**methylation of H3K9me2 by PHF8 to yield unmodified H3K9 (please refer to in-text reference Yu et al., 2010) which is then allowed to be acetylated by TIP60 to become H3K9ac. However, when we looked at each chromatin modification ie. H3K9ac, H3K14ac, and H3S10P, none was significantly altered in response to neuronal activity (please see additional data in the revised Figure 4). The only histone mark we observed to robustly increase in response to neuronal activity is the dual phosphoacetylation mark H3K9acS10P. The antibody we chose to study this phosphoacetyl mark is the ChIP grade ab12181 from Abcam (http://www.abcam.com/histone-h3-acetyl-k9-phospho-s10-antibody-chip-grade-ab12181.html) which is highly specific for the K9acS10P modification and does not bind to the H3K9ac or the H3S10P individual modifications. While the K9acS10P-specific ab12181 does **not** bind to H3K9ac without adjacent S10P or H3S10P without adjacent K9ac, antibodies against H3K9ac, on the other hand, are typically raised against a peptide with H3K9ac and unmodified S10, which may explain its ability to stain H3K9acS10P as well (Figure 3d, for example, would contain both H3K9ac and H3K9acS10P). The reason why we chose to address H3K9ac in the context of S10P is the importance of H3K9acS10P itself: this mark, although not very well studied in neurons, is indeed a major factor that drives transcriptional elongation, through the mechanism involving BRD4 and PTEFb described here:
  It is for this reason that we believe one of the most important results we are presenting is the direct modulation of H3K9ac, specifically the H3K9acS10P form, by PHF8-TIP60 (Figure 6, Figure 7, Figure 8a).
  With regard to the issue of the ability of complex to bind to either H3K9ac vs H3K9acS10P, while we are not in a position to exclude the possibility that the presence of S10P is detrimental to the activities of either PHF8 or TIP60, we would like to point out that there is evidence of exactly the opposite: in fact, the presence of S10P highly synergizes with H3 acetylation - TIP60’s purported action – (please see Mol Cell. 2000 Jun;5(6):905-15) while negatively regulating H3K9 methylation - PHF8’s purported action – (please see Nature, 406 (2000), pp. 593–599). These lines of evidence about S10P, which is itself a known neuronal activity marker, therefore increase the likelihood that the neuronal activity-dependent PHF8-TIP60 complex may be responsible for the formation of specifically the H3K9acS10P mark, keeping in mind that H3K9acS10P is a specific subset of the total pool of H3K9ac in a neuron, and it is this mark that is highly crucial for transcriptional elongation to occur in the mechanism described in this work.
3) Although the authors focus exclusively on the Arc gene, the data of which are convincing, I wonder why a more global genome-wide approach wasn't taken? To truly demonstrate that this complex globally controls activity-dependent transcription, I would need to see ChIP-seq experiments performed. Alternatively, the authors might consider significantly dampening their claims about the global role of this complex in transcription.
  We agree wholeheartedly with Reviewer #1 and have changed the title of the manuscript to “A neuronal activity-dependent dual-function chromatin modifying complex that increases Arc expression”
4) I am very glad that the authors present in vivo data, however, without presenting any biochemistry in the adult brain showing that PHF8 and TIP60 do indeed display increased binding after novel environment exposure, then I feel that the inclusion of these data are unnecessary.
  The in-vivo data has been excluded from the current manuscript, pending further biochemical work.
5) Lastly, it is my opinion that this manuscript is entirely too dense and needs to be streamlined significantly. As I said, the data presented are very nice, but inclusion of 13 figures to make their points seems excessive. I feel that a non-chromatin specialist would simply be lost.
  We have streamlined the manuscript to 10 relevant figures that have been reordered to represent the data more clearly and more succinctly.

### Reviewer #2

In "A dual function chromatin-modifying complex regulates neuronal activity-dependent gene transcription" the authors demonstrate for the first time the interaction of the demethylase PHF8 and the acetyltransferase TIP60 to regulate gene induction by neuronal activity. This interaction, which is consistently documented throughout the paper using diverse approaches, may have important consequences in regulating cognitive processes and may be a relevant target in neuropathologies with cognitive decline, such as intelectual disabilities and Alzheimer's disease. However, examination of a learning paradigm has not been within the scope of the present work. Several concerns should be addressed to make the conclusions stronger and to facilitate the reading: the high amount of figures and the variety of experiments requires an additional effort in the presentation of the data for a better comprehension.

1. The title of the manuscript suggest that this is a general mechanism, but so far only one activity-dependent gene has been examined in detail (Arc) and another one does not show the same behavior (Npas4 in Figure 8).
  We have included more data to show that the histone mark H3K9acS10P is specifically enriched in the Arc transcriptional start site in response to neuronal activity while it is not enriched in the TSS of a few other genes tested (including Synaptophysin, BDNF exon I, Txnip, JunB, etc.). have to agree with Reviewer #2 and have changed the title of the manuscript to “A neuronal activity-dependent dual-function chromatin modifying complex that increases Arc expression”
2. To some extent there is some inconsistency in presenting data in a balanced way for PHF8 and TIP60: for example, only a deletional dissection of TIP60 structure is shown in Figure 3, the concomitant quantification of H3K9ac is missing in Figure 4, in Figure 14 TIP60 induction is not shown whereas an overlay/correlation of PHF8 with Arc is lacking in later panels. We agree with Reviewer #2 and would like to apologize for these unintended inconsistencies. The domains of TIP60 are much more well-defined than those of PHF8 presently, and as such we are showing deletional dissection of TIP60 only. We have now shown the quantification for H3K9ac in Figure 4 (now called Figure 3e), and have decided to remove Figure 14 pending further biochemical work in-vivo.
3. It is concluded that TIP60 terminal chromo binding hinders association with PHF8 but construct E does not contain the N-terminus portion. Moreover, to conclude the effect of construct F it is important to know whether the presented results are representative of a series of duplicates. Although the chromo-domain seems to be inhibitory to association with PHF8 (comparing constructs F and C), the zinc finger domain seems to be important (hence explaining the decreased association in construct E). The results are indeed representative of a series of 2 biological replicates, and we have chosen to display the qualitatively cleaner Western Blot in Figure 3c. (now called Figure 2c)
4. The band intensities in Figure 3f are too uneven to make a conclusion of the experiment without a quantification.
  We have added a quantification panel to Figure 3f. (now called Figure 2f)
5. It is concluded from results in Figure 3h/i that the effect of transfecting both proteins is synergistic on acetylation of histone H3 but the difference between TIP60 and TIP60+PHF8 lanes is not obvious. In any case, as it is, it is relevant in the context of the present study to demonstrate a physical binding between H3K4me3 and H3K9ac that is enhanced in overexpressing conditions.
  While PHF8 and TIP60 overexpression both seem to have a modest effect in increasing *global* H3K9ac acetylation (currently Figure 2f), we noted that the increase in acetylation of H3K9 is specific to H3K4me3 positive histones (currently Figure 2h/i). Only a small percentage of all histones are H3K4me3 positive (a population that is known to be very tightly associated with transcriptional activation), and it is in this small subset of histones that acetylation of H3K9, K14, K18, and K27 are highly correlated (J Biol Chem. 2007 Feb 16;282(7):4408-16.). In other words, the existence of me3 in the K4 position predisposes the same histone to be acetylated at the K9, K14, K18, and K27 positions which is a transcriptionally permissive process, but little is known about how this is accomplished in neurons. Our work suggests that the enzymes responsible for the activity-dependent increase in H3K9 and K14 acetylation in neurons may be PHF8 (which binds to H3K4me3 and demethylates the adjacent H3K9me2→H3K9) and TIP60 (which acetylates H3K9 → H3K9ac). In Figure 2h/I, we performed a pull down of H3K4me3 and observed the level of acetylation at the K9 position in these H3K4me3-positive histones.
6. Check x-axis labels in Figure 4e: PHF8- is without overexpression of PHF8 but shows the lowest levels of dimethylation, the opposite of panel a and b; it is not obvious what it means F279S- (wild-type?).
  We sincerely apologize for this typo – indeed as the Reviewer pointed out, the x-axis labels were mismatched. This has now been fixed in Figure 4e (now called Figure 3e). F279S- and PHF8- both mean the neurons that are in the same microscopic field which were not expressing either mutant F279S or wild-type PHF8, hence, they served as control neurons for the quantitation of H3K9me2 and H3K9ac.
7. The authors indicate that "various acetylation sites tested, we observed that H3K9acS10p was the most responsive to synaptic activity". It is important to show the tested marks, and more considering that the responsiveness of H3K9acS10p may be by the sole modification of Serine 10 as the antibody may recognizes either the acetylation or phosphorylation. Because the basal signal for the immunohistochemistry (Figure 14) is neglectable, it is reasonable to think that the antibody only recognizes the phosphorylation change, which is virtually absent in basal state, therefore compromising the link with the acetylation and the enzymatic function of PHF8-TIP60. We have included the results from high-content neuronal screening of the histone marks tested: we initially looked at H3K9ac, H3K14ac, and H3S10P, but although we saw some trends, none of these marks was strongly responsive to synaptic activity. As Figure 3 has shown, PHF8-TIP60 are capable of acetylating H3K9, but to our surprise, the histone mark that is specifically and robustly increased by neuronal activity is in fact H3K9acS10P, which tells us that although K9ac is important, the activity-dependent modification of K9 by PHF8-TIP60 happens very specifically in conjunction with S10 phosphorylation (in other words, S10P has to be present first). As discussed in the text, the presence of H3S10P does not impede further acetylation of the same N-terminal H3). The antibody we used to detect H3K9acS10P is highly specific for histones that must have both K9ac and the adjacent S10P (where both the phosphorylation and acetylation marks must exist in tandem). The interpretation of this data is that PHF8-TIP60 modulates H3 acetylation in the context of S10P, which is itself a well-known marker of synaptic activity, in order to produce H3K9acS10P, which is an important chromatin mark associated with transcriptional elongation (Cell. 2009 Sep 18;138(6):1122-36.).
8. Figure 8 contains a highly important message (the binding of TIP60 and PHF8 at the Arc promoter) that is worthy to indicate in the main text. Is it possible to add the profile of H3K9me2 in panel a?
  We agree with the Reviewer and have decided to make these important events a focus in the article by incorporating it into the title and the abstract. The profile of H3K9me2 at the Transcriptional Start Sites of various neuronal genes is now shown in Figure 8 to echo the profile seen in high-content immunohistochemistry screening (Figure
9. The introduction of the mass spectrometry in HEK293 cells breaks the flow of the manuscript in its present form. Apart from providing a testable candidate in Figure 12, it is not obvious the main message of this experiment: is the overlap significant? Consider to present earlier this piece of information, before the work with neuronal cultures. In addition, nucleolin appears as a common interacting partner but Figure 2 indicates lack of colocalization of PHF8-nucleolin. The IP-MS data is now presented as Figure 8 in the text to improve the flow through the manuscript. The introduction of PSF as a common binding partner between PHF8 and TIP60 introduces the super-resolution microscopy data presented in the final Figures 9 and 10. The Nucleolin construct used was overexpressed and may not have indicated the physiological behavior of endogenous Nucleolin and Figure 2 has therefore been excluded.
10. It is intriguing that no signal is observed in the basal state for either PHF8 and TIP60 in Figures 13 and 14 as they can be expressed in brain. A quantitative RT-PCR may solve this issue.
  Though we see staining at the level of single cells at high magnification, we agree with the Reviewer that the immunohistochemistry signal shown in these figures are not entirely representative. We have therefore decided to exclude Figures 13-14.

### Minor points:

1. In the Introduction it is said that "stimulus-dependent rapid gene induction... is heavily dependent on dynamic chromatin modification". Be more precise, as certain aspects of epigenetic-based regulation are more complex as originally proposed in cognitive and activity-dependent gene expression (see the review by Lopez-Atalaya and Barco in TiG). In addition, the provided references focus on the histone-code hypothesis rather than review the evidences supporting the present work; more recent reviews may be more appropriate (for example, Sweatt's commentary in Neuron).
  We thank the Reviewer for these highly relevant and much more supportive references. We have included and discussed these references in the text.
2. Explain better the P-LISA technique, as readers may be more familiar with FRET-based techniques. This approach is highly relevant to demonstrate the interaction PHF8-TIP60 and it is not even mentioned in the Methods section.
  We thank the Reviewer for picking up on this crucial point. We have included a comprehensive Methods section on P-LISA and have cited the proper references.
3. Show the DAPI channel alone in Figure 1c to verify that do not significantly overlap with the labeling of PHF8 and S5P.
  The DAPI-only channel has now been included in Figure 1c.
4. It is said that the mutation F279S of PHF8 is clinically relevant. Specify a reference. Is it a dominant negative? An answer will clarify the results of Figure 1i.
  The appropriate references for the clinical mutation of F279S which renders the enzyme demethylase-inactive has been included.
5. In Figure 3g a loading control is missing.
  The loading control for Figure 3g is now included.
6. The low intensity in the staining of both modified histones in Figure 4c may be misleading. The LUT (Look-Up Table) for Figure 4 has been standardized. Changes in the intensity of fluorescence signal reflects changes in the concentration of the respective antibodies.
7. Add axis labels in Figure 5. Axis labels have been added to Figure 5.
8. References to Figure 2e is not correct in the section "PHF8 and TIP60 are members of a dual function complex, etc" and to Figure 7b-c when talking about H3K9acP10s reduction.
  Figure 2e shows that PHF8 and TIP60 increase H3K9 acetylation in HEK293 cells and have been rephrased as such. Figure 7b-c has been replaced by Figure 7a.
9. 9. Consider to merge Figure 13 and 14 (or to remove Figure 13).
  Figure 13 and 14 have been excluded from the manuscript.
